# Comparative Sequence and Structure Analysis Reveals the Conservation and Diversity of Nucleotide Positions and Their Associated Tertiary Interactions in the Riboswitches

**DOI:** 10.1371/journal.pone.0073984

**Published:** 2013-09-05

**Authors:** Sri D. Appasamy, Effirul Ikhwan Ramlan, Mohd Firdaus-Raih

**Affiliations:** 1 School of Biosciences and Biotechnology, Faculty of Science and Technology, Universiti Kebangsaan Malaysia, UKM Bangi, Selangor, Malaysia; 2 Department of Artificial Intelligence, Faculty of Computer Science and Information Technology, University of Malaya, Kuala Lumpur, Malaysia; 3 Institute of Systems Biology, Universiti Kebangsaan Malaysia, UKM Bangi, Selangor, Malaysia; University of Edinburgh, United Kingdom

## Abstract

The tertiary motifs in complex RNA molecules play vital roles to either stabilize the formation of RNA 3D structure or to provide important biological functionality to the molecule. In order to better understand the roles of these tertiary motifs in riboswitches, we examined 11 representative riboswitch PDB structures for potential agreement of both motif occurrences and conservations. A total of 61 unique tertiary interactions were found in the reference structures. In addition to the expected common A-minor motifs and base-triples mainly involved in linking distant regions the riboswitch structures three highly conserved variants of A-minor interactions called G-minors were found in the SAM-I and FMN riboswitches where they appear to be involved in the recognition of the respective ligand’s functional groups. From our structural survey as well as corresponding structure and sequence alignments, the agreement between motif occurrences and conservations are very prominent across the representative riboswitches. Our analysis provide evidence that some of these tertiary interactions are essential components to form the structure where their sequence positions are conserved despite a high degree of diversity in other parts of the respective riboswitches sequences. This is indicative of a vital role for these tertiary interactions in determining the specific biological function of riboswitch.

## Introduction

Riboswitches are RNA regulatory elements that affect gene expression by binding to specific, small-molecule metabolites [1], [2], [3]. They are typically found in the 5′-UTR in bacteria although some have been found residing in the introns or 3′-UTR of eukaryotic transcripts [4]. Currently, there are over twelve different classes of riboswitches that respond to a diverse array of metabolites ranging from simple purine nucleotides to complex enzymatic cofactors [5]. The most common strategy employed by riboswitches to regulate gene expression involve the formation of a rho-independent terminator helix that results in transcription termination, or formation of a helix, which sequesters the Shine-Dalgarno sequence thereby preventing translation from taking place [6]. Other novel gene regulation mechanisms utilized by riboswitches include self-cleaving activity by the glucosamine-6-phosphate (GlcN6P) riboswitch [7] and cooperative binding by glycine riboswitches [8], [9].

Riboswitches fold into complex three-dimensional structures in order to perform their regulatory functions. Structural analyses on riboswitches have revealed that they are assembled from motifs or structural elements that have been observed in other functional RNA molecules [10], [11]. Examples include base triples, kink-turns, kissing-loops and ribose zippers. The occurrence of base triple interactions has been previously reported in riboswitches [12], [13]. Base triples can result in widened grooves within double stranded RNA or they mediate tertiary interaction involving a third strand. For instance, a UAU triple results in the widening of the RNA groove required for binding of arginineamide in many retroviral protein-RNA complexes such as HIV-1 [14] and HIV-2 [15] argininamide-TAR RNA complexes, the BIV Tat-TAR RNA [16] and the HIV-1 Rev RRE RNA aptamer [17] complexes.

In this paper, we present a survey to investigate the conservation of tertiary structural motifs at the nucleotide sequence level. Our analysis covered seven different tertiary motifs, namely base-triples, A-minor motifs, ribose zippers, loop-loop interactions, loop-loop receptors, kink-turns and pseudoknots. Our results focus particularly on the conservation of base triples at sequence level for occurrences that were detected in crystallographic structures from Protein Data Bank (PDB) [18].

## Materials and Methods

### Datasets

Seventy one riboswitch structures solved by X-ray crystallography to a minimum resolution of 3Å were downloaded from the PDB. The list of downloaded structures included ten structurally different classes of riboswitches: (i) purine riboswitch [19]; (ii) SAM-I riboswitch [20]; (iii) SAM-II riboswitch [13]; (iv) SAM-III riboswitch [21]; (v) PreQ1 riboswitch [22]; (vi) Lysine riboswitch [23]; (viii) Flavin mononucleotide riboswitch [24]; (viii) Thiamine pyrophosphate riboswitch [25]; (ix) Magnesium riboswitch [26]; and (x) c-di-GMP riboswitch [27] (Table S1).

### Structure Annotation

The seventy one structures were then annotated for tertiary motifs using a combination of manual compilation from literature and automated annotation using available computer programs. The computer program NASSAM [28], [29] (http://mfrlab.org/grafss/nassam) was used to search for: i) A-minor motifs [30]; ii) base-triples [29], [31]; iii) ribose zippers [32]; and iv) kink-turns [33] in the structures that were retrieved from the PDB. The distance tolerance was set to 60% in order to retrieve all possible arrangements available in the search database provided. All hits returned by NASSAM were then visualized manually. For base triples, only hits that were planar interactions consisting of at least two hydrogen bond interactions per base pair were selected for further analysis. Loop-loop interactions [34], loop-loop receptors [35] and pseudoknots [36] were identified by visual inspection using RNAVIEW [37].

### Sequence Alignments and Structure Superpositions

**Figure 1:**
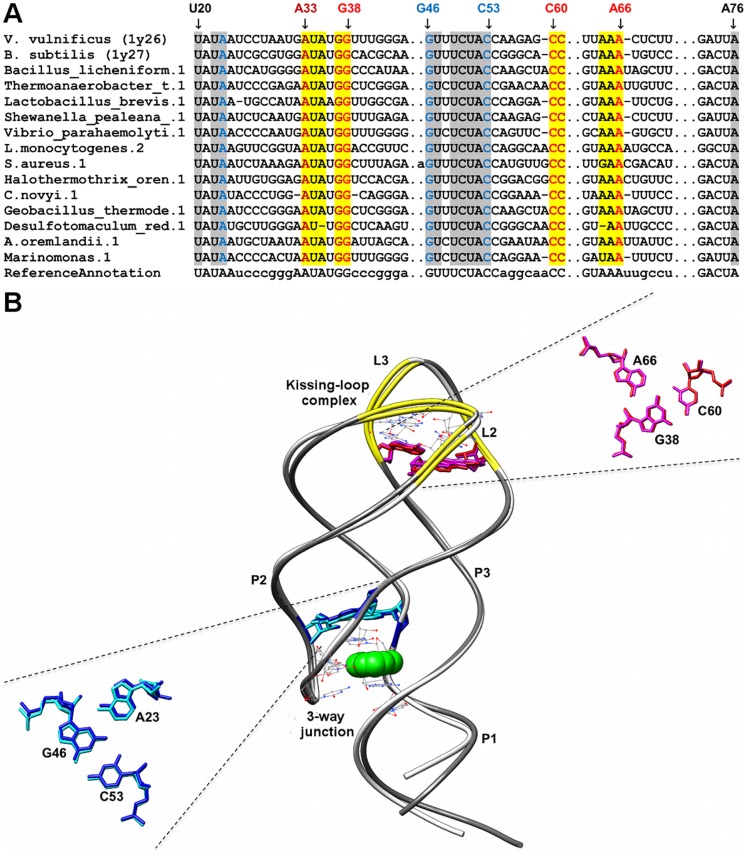
Sequence alignment and annotated tertiary motifs in purine riboswitches. (A) Sequence alignment of purine riboswitches where gray shaded columns indicate the highly conserved nucleotide positions. Other shaded columns correspond to regions involved in tertiary interactions (yellow: kissing-loop and brown: A-minor motifs). For clarity, not all tertiary motif positions are numbered. (B) Structural superposition between *V. vulnificus* purine riboswitch [19] (PDB ID = 1y26/dim gray backbone) and *B. subtilis* purine riboswitch [19] (PDB ID = 1y27/white backbone). The ligand is represented as spheres and the highly conserved nucleotides are in wire representation. Magnifications of the superposed triples using the numbering for 1y27 are presented in blue (1y27), cyan (1y26), red (1y27), and magenta (1y26).

**Figure 2:**
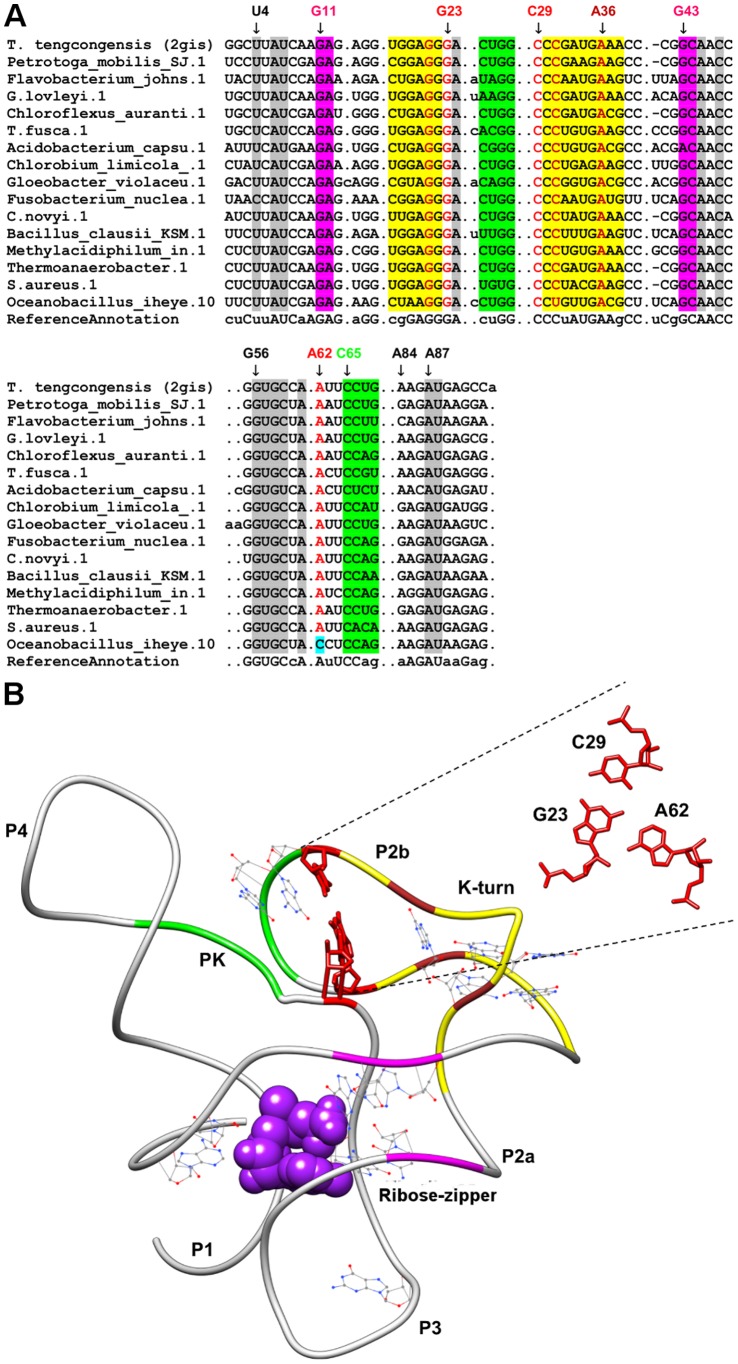
Sequence alignment and annotated tertiary motifs in SAM-I riboswitches. (A) Sequence alignment of SAM-I riboswitches where gray shaded columns indicate the highly conserved nucleotide positions. Other color shaded columns indicate regions involved in tertiary interactions (green: pseudoknot, yellow: kink-turn, magenta: ribose-zipper and brown: A-minor). For clarity, not all tertiary motif positions are numbered. Base-triple positions that are not conserved are in cyan shaded columns. (B) Tertiary structure of *T. tengcongensis* SAM-I riboswitch [20] (PDB ID = 2gis). The ligand is represented as spheres and the highly conserved nucleotides are in wire representation. Magnification of the triple using numbering for 2qis is presented in red (A23-G23-C29).

**Figure 3:**
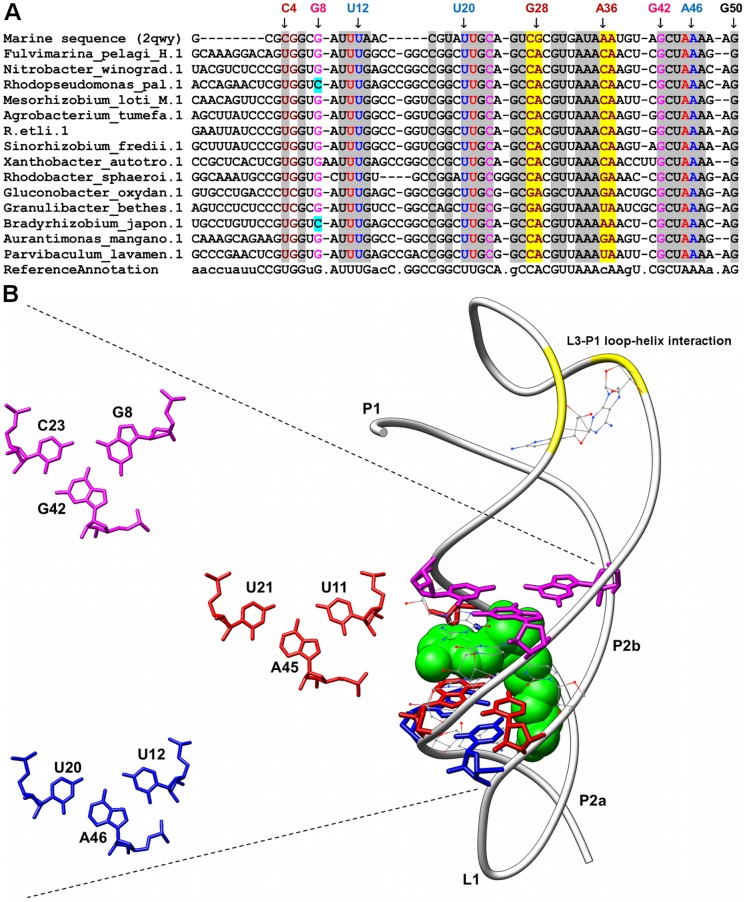
Sequence alignment and annotated tertiary motifs in SAM-II riboswitches. (A) Sequence alignment of SAM-II riboswitches where gray shaded columns indicate the highly conserved nucleotide positions. Other color shaded columns indicate regions involved in tertiary interactions (brown: A-minor and yellow: ribose-zipper). For clarity, not all tertiary motif positions are numbered. Base-triple positions that are not conserved are in cyan shaded columns. (B) Tertiary structure of SAM-II riboswitch [13] (PDB ID: 2qwy). The ligand is represented as spheres and the highly conserved nucleotides are in wire representation. Magnification of the triples using numbering for 2qwy are presented in red (G8-C23-G42), magenta (U11-U21-A45), and blue (U12-U20-A46).

**Figure 4:**
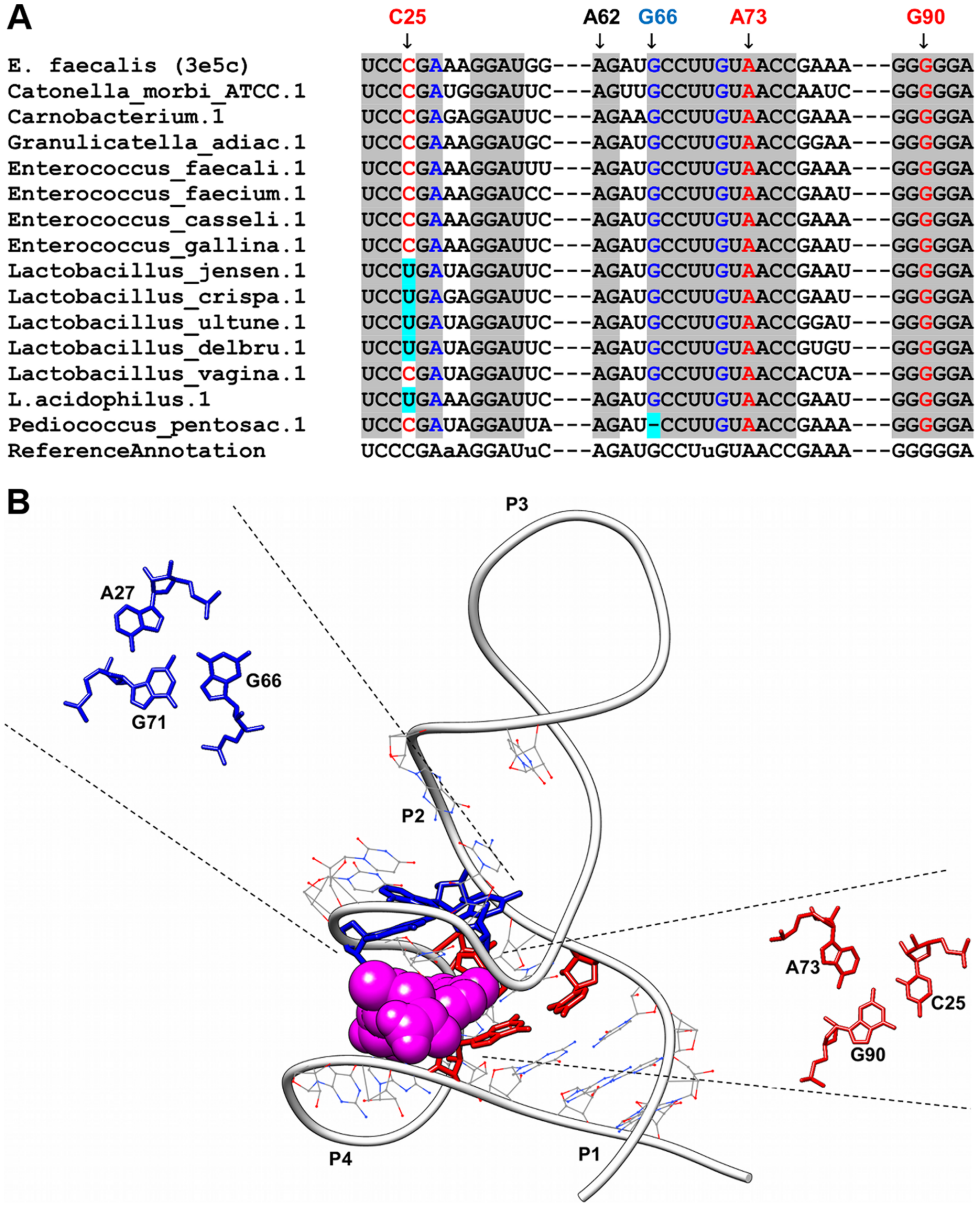
Sequence alignment and annotated tertiary motifs in SAM-III riboswitches. (A) Sequence alignment of SAM-III riboswitches where gray shaded columns indicate the highly conserved nucleotide positions. For clarity, not all tertiary motif positions are numbered. Base-triple positions that are not conserved are in cyan shaded columns. (B) Tertiary structure of the *E. faecalis* SAM-III riboswitch [21] (PDB ID = 3e5c). The ligand is represented as spheres and the highly conserved nucleotides are in wire representation. Magnification of the triples using numbering for 3e5e are presented in red (A73-G90-C25) and blue (A27-G66-G71).

**Figure 5:**
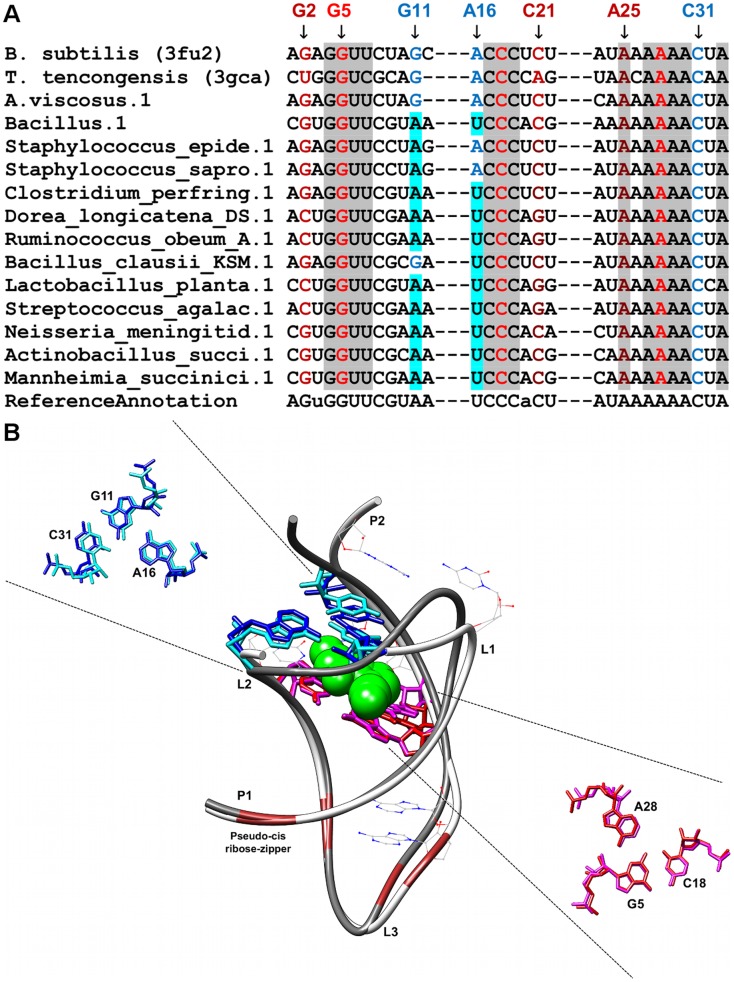
Sequence alignment and annotated tertiary motifs in preQ1 riboswitches. (A) Sequence alignment of preQ1 riboswitches where gray shaded columns indicate the highly conserved nucleotide positions. Brown shaded columns indicate regions involved in the pseudo-cis ribose-zipper interaction. For clarity, not all tertiary motif positions are numbered. Base-triple positions that are not conserved are in cyan shaded columns. (B) Structural superposition between *B. subtilis* preQ1 riboswitch [22] (PDB ID = 3fu2/white backbone) and *T. tengcongensis* preQ1 riboswitch [42] (PDB ID = 3gca/dim gray backbone). The ligand is represented as spheres and the highly conserved nucleotides are in wire representation. Magnifications of the superposed triples using the numbering for 3fu2 are presented in blue (3fu2), cyan (3gca), red (3fu2) and magenta (3gca).

**Figure 6:**
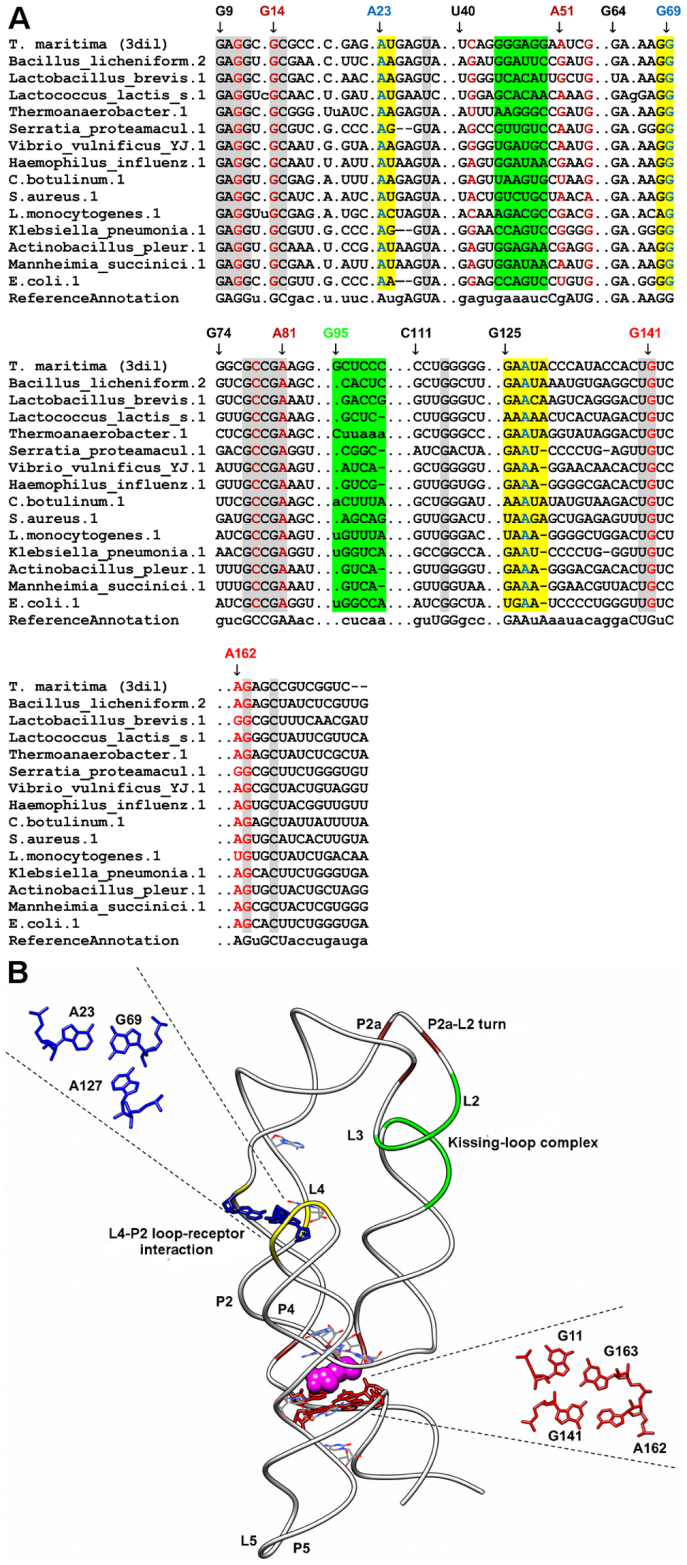
Sequence alignment and annotated tertiary motifs in lysine riboswitches. (A) Sequence alignment of lysine riboswitches where gray shaded columns indicate the highly conserved nucleotide positions. Other color shaded columns indicate regions involved in tertiary interactions (brown: A-minor, yellow: loop-receptor interaction and green: kissing-loop complex). For clarity, not all tertiary motif positions are numbered. Base-triple positions that are not conserved are in cyan shaded columns. (B) Tertiary structure of *T. maritima* lysine riboswitch [23] (PDB ID = 3dil). The ligand is represented as spheres and the highly conserved nucleotides are in wire representation. Base triple (A127-A23-G69) and base quadruple (G11-G163-G141-A162) interactions are presented in blue and red respectively.

**Figure 7:**
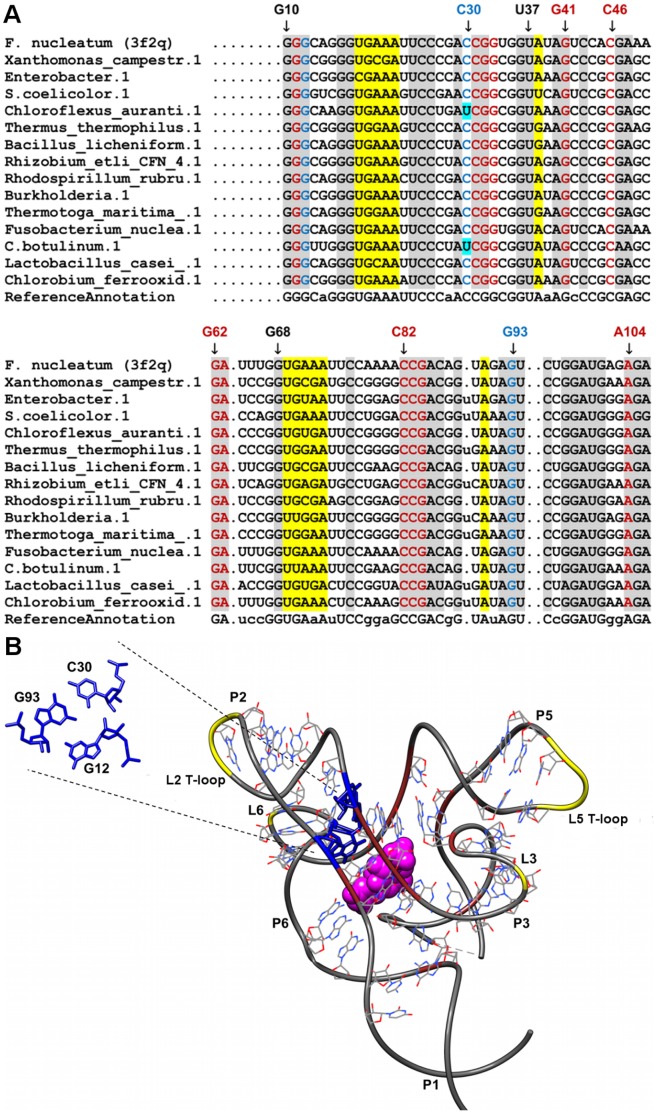
Sequence alignment and annotated tertiary motifs in FMN riboswitches. (A) Sequence alignment of FMN riboswitches where gray shaded columns indicate the highly conserved nucleotide positions. Other color shaded columns indicate regions involved in tertiary interactions (brown: A-minor and yellow: T-loop). For clarity, not all tertiary motif positions are numbered. Base-triple positions that are not conserved are in cyan shaded columns. (B) Tertiary structure of *T. maritima* FMN riboswitch [24] (PDB ID = 3f2q). The ligand is represented as spheres and the highly conserved nucleotides are in wire representation. Magnification of the triple using numbering for 3f2q is presented in blue (G12-C30-G93).

**Figure 8:**
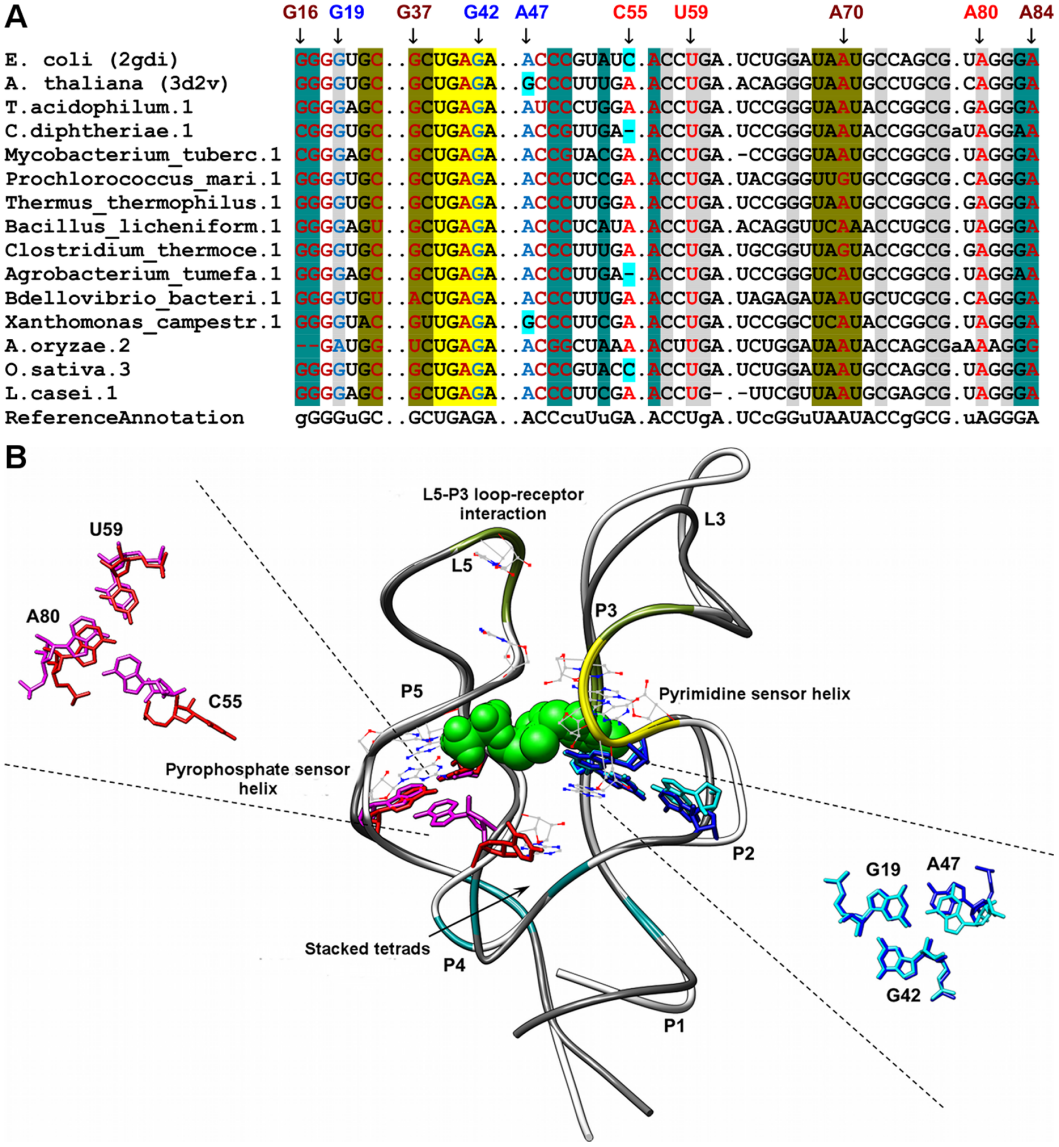
Sequence alignment and annotated tertiary motifs in TPP riboswitches. (A) Sequence alignment of TPP riboswitches where gray shaded columns indicate the highly conserved nucleotide positions. Other color shaded columns correspond to regions involved in tertiary interactions (teal: stacked tetrads, brown: A-minor, dark yellow: loop-loop receptor, and yellow: T-loop). For clarity, not all tertiary motif positions are numbered. Base-triple positions that are not conserved are in cyan shaded columns. (B) Structural superposition between *E. coli* TPP riboswitch [25] (PDB ID = 2gdi/white backbone) and *A. thaliana* TPP riboswitch [43] (PDB ID = 3d2v/dim gray backbone). The ligand is represented as spheres and the highly conserved nucleotides are in wire representation. Magnifications of the superposed triples using numbering for 2gdi are presented in blue (2gdi), cyan (3d2v), red (2gdi) and magenta (3d2v).

**Figure 9:**
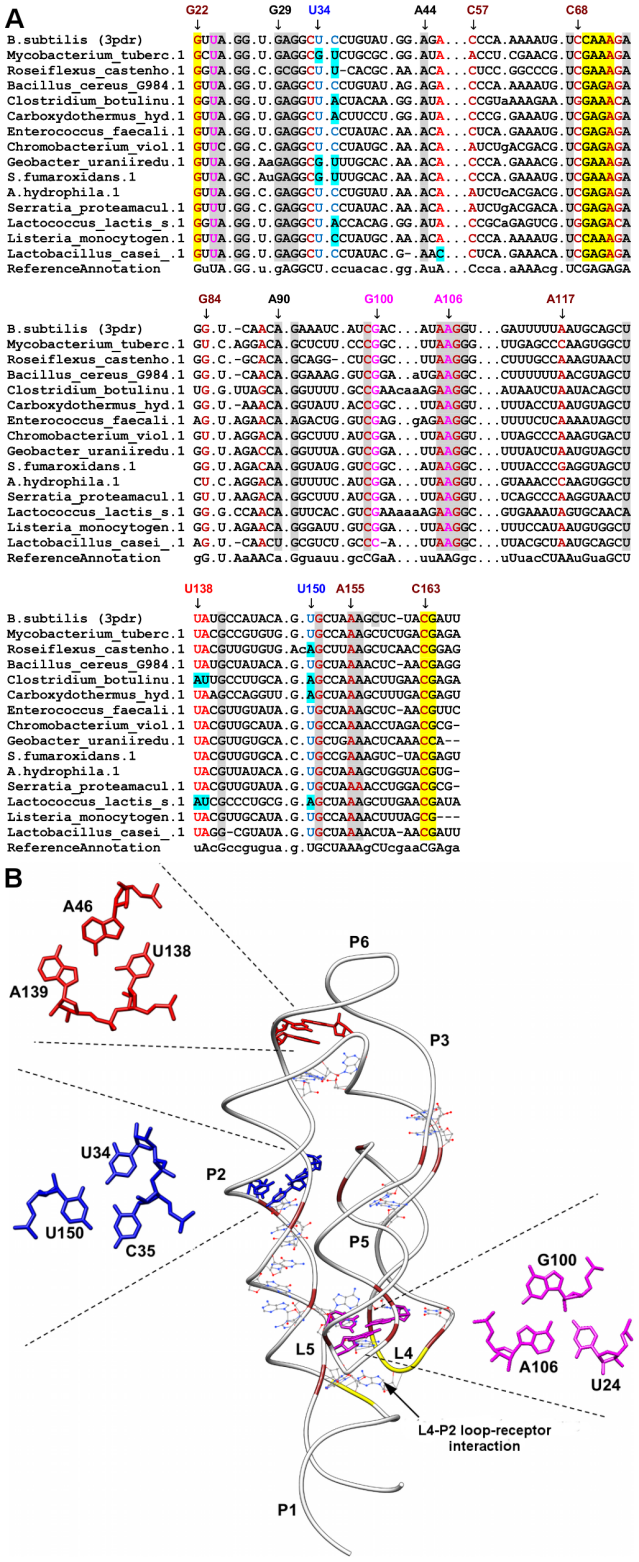
Sequence alignment and annotated tertiary motifs in Mg^2+^ riboswitches. (A) Sequence alignment of magnesium riboswitches where gray shaded columns indicate the highly conserved nucleotide positions. Other color shaded columns indicate regions involved in tertiary interactions (brown: A-minor and yellow: loop-receptor interaction). For clarity, not all tertiary motif positions are numbered. Base-triple positions that are not conserved are in cyan shaded columns. (B) Tertiary structure of *B. subtilis* Mg^2+^ [26] riboswitch (PDB ID = 3pdr). The ligand is represented as spheres and the highly conserved nucleotides are in wire representation. Magnifications of the triples using numbering for 3pdr are presented in red (A46-U138-A139), blue (U34-C35-U150) and magenta (U24-G100-A106).

**Figure 10:**
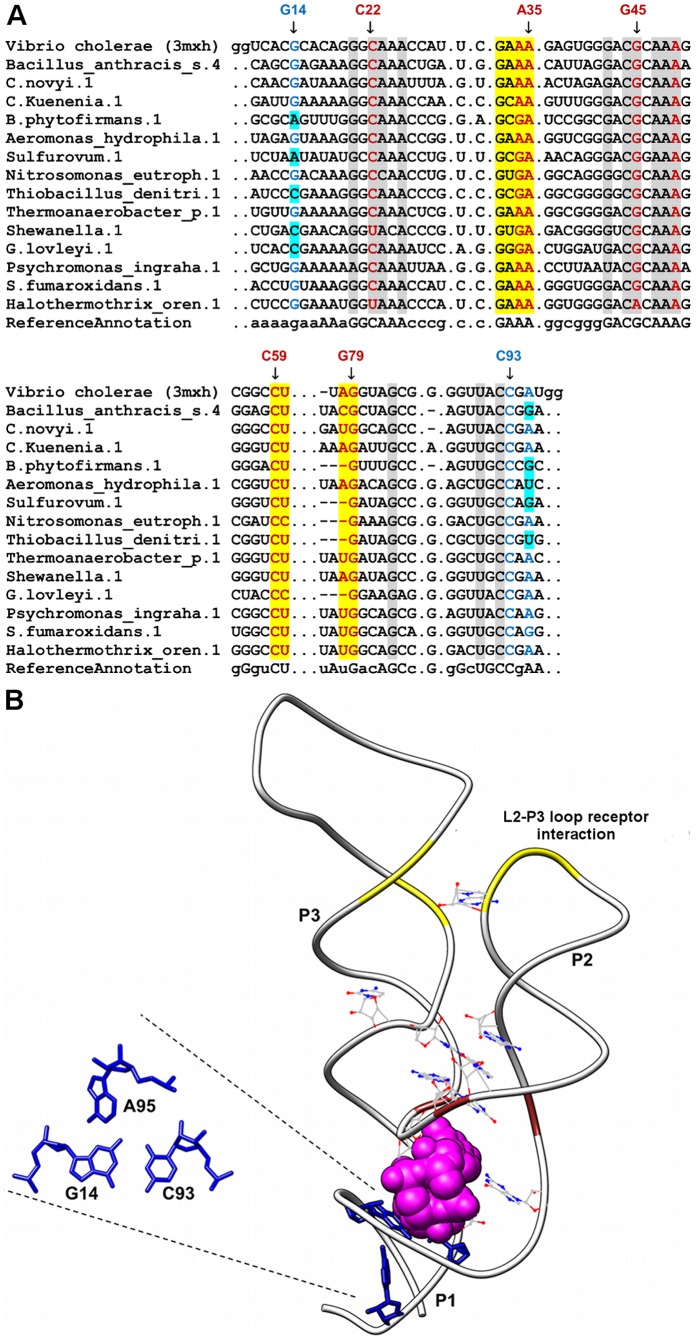
Sequence alignment and annotated tertiary motifs in c-di-GMP riboswitches. (A) Sequence alignment of c-di-GMP riboswitches where gray shaded columns indicate the highly conserved nucleotide positions. Other color shaded columns indicate regions involved in tertiary interactions (brown: A-minor and yellow: loop-receptor interaction). For clarity, not all tertiary motif positions are numbered. Base-triple positions that are not conserved are in cyan shaded columns. (B) Tertiary structure of *V. cholerae* c-di-GMP riboswitch [27] (PDB ID = 3mxh). The ligand is represented as spheres and the highly conserved nucleotides are in wire representation. Magnification of the triple using numbering for 3mxh is presented in blue (A95-G14-C93).

Rfam [38] seed alignments were downloaded for the respective riboswitches. INFERNAL 1.0 [39] was used to align the riboswitch sequences extracted from PDB structures to the Rfam seed alignments based on the covariation model built from the seed alignment. JalView [40] was used for visual examination of the alignments. Nucleotide positions with more than 95% sequence conservation in the Rfam seed alignments were identified and highlighted as gray shaded columns in Figures 1, 2, 3, 4, 5, 6, 7, 8, 9, 10. The complete Rfam alignments are provided as Supplementary materials (Figures S1, S2, S3, S4, S5, S6, S7, S8, S9, S10). Structure superpositions were done using the program Chimera [41] through the MatchMaker function. The structures analysed were: (i) *V. vulnificus* (1y26) [19] and *B. subtilis* (1y27) [19] for purine riboswitch, ii) *B. subtilis* (3fu2) [22] and *T. tengcongensis* (3gca) [42] for preQ1 riboswitch and iii) *E. coli* (2gdi) [25] and *A. thaliana* (3d2v) [43] for TPP riboswitch. Full species names and corresponding database identifier codes of the sequences used in the alignments are provided in Table S1.

## Results and Discussion

### Conservation of Base Triples in Riboswitches

A total of nineteen base-triples were found in the reference structures representing ten riboswitch classes (Table 1). Out of the nineteen base triples, thirteen of them contain a Watson-Crick pair, either a G-C or A-U whereas the remaining triples involve pairings between non-canonical base pairs. The AGC amino-N3, N1-amino Watson-Crick triple was found to be most common triple in our annotation with seven occurrences in different riboswitch structures (Table 1). Most of the triples identified involve interactions between different secondary structure elements of RNA namely helix-hairpin loop interactions and helix-junction interactions.

**Table 1:** List of base-triples identified by NASSAM in riboswitches.

PDBID	Base triple and associated secondary structure interaction	Riboswitch class
1y26	A23-**G46-C53**Interaction between junctions (J1–2 & J2–3)	Purine
1y26	A66-**G38-C60**Hairpin loop – hairpin loop interaction (L2 & L3)	Purine
2gis	A62-**G23-C29**Helix – junction interaction (P2b & J3-4)	SAM-I
2qwy	G8-**G42-C23**Helix – hairpin loop interaction	SAM-II
2qwy	U11-**A45-U21**Helix – hairpin loop interaction (P2b & L1)	SAM-II
2qwy	U12-**A46-U20**Helix – hairpin loop interaction (P2b & L1)	SAM-II
3e5c	A27-G71-G66Helix – junction interaction (P2 & J3-2)	SAM-III
3e5c	A73-**G90-C25**Helix – junction interaction (P1 & J2–4)	SAM-III
3fu2	A16-**G11-C31**Helix – hairpin loop interaction (P2 & L2)	PreQ1
3fu2	A28-**G5-C18**Helix – hairpin loop interaction (P1 & L3)	PreQ1
3dil	A23-G69-A127Helix – hairpin loop interaction (P2 & L4)	Lysine
3dil	G141-A162-G163Helix – helix interaction (P1 & P5)	Lysine
3f2q	G12-**G93-C30**Junction – helix – hairpin loop interaction (J1-2, P2, L5)	FMN
2gdi	G19-G42-A47Helix – junction interaction (P2 & J3-2)	TPP (prokaryotic)
3d2v	A43-**A68-U47**Helix – junction interaction (P4 & J2–4)	TPP (eukaryotic)
3pdr	**U24**-**A106**-G100Bulged-out residue – helix interaction (P2 & P5)	Magnesium
3pdr	A46-U138-A139Interaction within junction (J2–6)	Magnesium
3pdr	C35-U34-U150Bulged-out residue – helix interaction (P2)	Magnesium
3mxh	A95-**G14-C93**Bulged-out residue – helix interaction (P1)	Cyclic di-GMP

Watson-Crick base-pairs are in bold face and the secondary structure elements are abbreviated as follows; P = Paired region, J = junction and L = loop.

Only six base triples were fully conserved in all the sequences analyzed for the respective riboswitch classes, two of each occurring in purine (A23-G46-C53 and A66-G38-C30) and SAM-II (U11-A45-U21 and U12-A46-U20) riboswitches respectively, one occurring in the preQ1 riboswitch (G5-C18-A28) and the final base triple is found in the lysine riboswitch (A127-A23-G69). The first AGC triple (A23-G46-C53) in the purine riboswitch (PDBID: 1y26) is involved in the formation of the ligand-binding pocket whereas the second AGC triple (A66-G38-C60) is involved in joining the terminal loops L2 and L3 (Figure 1). In the SAM-II riboswitch (PDBID: 2qwy), the stacked UAU Hoogsteen, Watson-Crick triples are involved in the ligand-binding pocket formation (Figure 3). The AGC triple (G5-C18-A28) in the preQ1 riboswitch (PDBID: 3fu2) is also involved in the formation of ligand-binding pocket whereas the AAG triple (A127-A23-G69) in the lysine riboswitch (PDBID: 3dil) is involved in a long distance tertiary interaction where it links pentaloop L4 to helix P2 (Figure 6).

The remaining base triples were observed to be highly conserved in sequence although not for all sequences in their respective riboswitch class with the exception of three base triples (A16-G11-C31, C35-U34-U150, and A95-G14-C93) occurring in the preQ1, Mg^2+^ and cyclic-di-GMP riboswitches respectively. In the preQ1 riboswitch [22], the ligand is sandwiched between two AGC triples with A16-G11-C31 triple being positioned above the ligand. Two base positions in this base triple are variable as adenine can substitute for G11 and uracil can substitute for A16. Both the substitutions occur concurrently which might lead to the formation of a possible alternative base triple interaction (A11-U16-C31). The last two non-conserved base-triples (C35-U34-U150 and A95-G14-C93) are examples of interactions involving a helix and a bulged-out residue from the same helix.

### Annotation and Conservation of Other Tertiary Interactions

We found an additional forty two RNA tertiary interactions (excluding base-triples) in our annotation. The complete list of the annotated tertiary interactions in riboswitches is provided in the Table S2. Large riboswitch structures such as Mg^2+^ and lysine riboswitches [23], [26] were observed to have more diverse tertiary interactions compared to the smaller riboswitches. However, base-triples and A-minor motifs occur frequently in most of the riboswitch classes annotated in this study and the occurrences of these interactions were not exclusive to a particular riboswitch class. The A-minor interactions were found to be the most frequent tertiary motif with twenty four interactions found. Both the type I and type II A-minor motifs [30] have an almost equal distribution in the riboswitches studied (Type I; 50%, type II; 46% of total A-minor motifs found). We did not observe any type III A-minor motifs in our annotation although a lone type-0 A-minor motif was found within the kink-turn structure in the SAM-I riboswitch [20] (Figure 2). Among the 24 A-minor motifs found, 25% interact with the GC receptor base pair, 71% with CG, and 4% with UA. These A-minor motifs were found to be highly conserved as they are largely involved in multiple long-range base interactions or they help to stabilize other more complex tertiary interactions such as kink-turn [33] or tetraloop-receptor interactions [44]. Exceptions were observed in the FMN riboswitch where they appear to play functional roles by helping to recognize the phosphate oxygens of FMN [24].

Ribose zippers [32] are the third most abundant tertiary interaction, with six occurrences in our annotation. Three of them were found to be part of loop-loop receptor interactions where they help to pack the loop and receptor nucleotides together [32]. These interactions are moderately conserved except for a highly conserved canonical ribose-zipper occurring in SAM-I riboswitches (Figure 2).

Two of the four identified loop-loop receptor interactions belong to the tetraloop-receptor type where they are observed in Mg^2+^ [26] and c-di-GMP riboswitches [27]. A tetraloop-receptor variant was observed in TPP riboswitch [25] in which a UNRN tetraloop docks to the receptor helix. Another interesting variant was found only in the lysine riboswitch [23] as this tertiary interaction involves a pentaloop interacting with the receptor helix.

Finally, four loop-loop interactions were found in our annotation. Loop-loop interactions involving complementary sequences, or more commonly known as kissing-loop interactions [34] were observed in purine and lysine riboswitches [19], [23]. Both these interactions show differing extent of conservation in our alignments. On the one hand, the kissing-loop interaction in the purine riboswitch (Figure 1) is highly conserved, while on the other, this interaction is only sparsely available. The final two loop-loop interactions were found in the FMN riboswitch [24] involving two highly conserved T-loop motifs in L5 and L2.

### Conservation of Specific Nucleotides in Riboswitches

A total of 236 nucleotide positions were found to have more than 95% sequence conservation in the alignments (Figures 1, 2, 3, 4, 5, 6, 7, 8, 9, 10; Figures S1, S2, S3, S4, S5, S6, S7, S8, S9, S10; Table S3) carried out. Most of the conserved nucleotides are involved in either ligand binding pocket formation or they form specific tertiary interactions that have both functional and structural roles. In addition, nucleotides in the single-stranded regions such as hairpin loops, internal loops and junctions provide the means for long distance interactions where they help to bring distant regions in the riboswitch structure into close contact. Junction loops play significant roles in riboswitch structures by providing unpaired nucleotides that help to recognize the ligand, interact with each other or with base pairs in the surrounding helices through extensive hydrogen-bonding network to form higher-order base interactions such as base triples or base quadruples which then form the ligand binding pocket or help to stabilize the binding of ligand to the RNA.

The FMN riboswitch [24] contains the highest number of highly conserved nucleotides in our alignments with 45 nucleotides (Figure 7). This riboswitch consist of six stem-loop structures where the conserved nucleotides are scattered unevenly among the various stem-loop structures. Two pairs of important tertiary interactions exist in this riboswitch; loop-loop interactions (L2-L6 and L3-L5) and loop-helix (L6-P2 and L3-P5) interactions that allow the FMN riboswitch to adopt a ‘butterfly’ like scaffold [24]. Therefore the steric requirement for the formation of this scaffold might account for the high conservation of these nucleotides. This is further supported by our observation where there is an absence of conserved nucleotides in the P4 stem-loop structure (i.e., this stem-loop structure is not involved in any structural interactions with the other stem-loops in this riboswitch [24]).

### Purine Riboswitches

We identified two AGC amino-N3, N1-amino Watson-Crick triples in the purine riboswitch investigated in [19]. The first triple, A23-G46-C53 (Figure 1), is one of the five base triples previously identified to be involved in the formation of the ligand binding pocket in purine riboswitches [19]. In the available structures, this interaction forms the uppermost base-triple in the ligand binding pocket and therefore when stacked together with the other triples, contribute to the stabilization of RNA-ligand interaction. All the five base triples were found to be highly conserved in the alignments. The nucleotide in sequence position Y74 of the recognition triple (U22-U51-Y74) was found to be variable as the identity of this nucleotide is important in distinguishing whether an adenine or a guanine ligand would be recognized by the riboswitch via Watson-Crick base-pairing (i.e., cytosine for guanine recognition and uracil for adenine recognition [44]). The second AGC triple, (A66-G38-C60) (Figure 1) is involved in the kissing-loop interaction between L2 & L3 [19] and is part of a base tetrad (A33-A66-C60-A38) where the non-canonical base pair, A33-A66 interacts with the minor groove of Watson-Crick base pair G38-C60. Furthermore, another base tetrad is formed between the hairpin loops (U34-A65-C61-G37) and together they help to anchor the kissing interaction between the terminal loops (L2 & L3) that eventually result in parallel alignment of stems P2 and P3. The base quadruples are stabilized by an A-minor interaction formed between A33 of the first base quadruple and the G-C pair (G37-C61) in the second base quadruple. This kissing interaction has been shown to be critical for preorganization of the global fold of the riboswitch prior to ligand binding, without which ligand binding would not take place [45], [46].

The majority of the highly conserved nucleotides in this riboswitch are found in the terminal loops and the core region [47], [48] that contains the three-way junction. These nucleotides are highly conserved because they are important for either the formation of triples which constitute the purine binding pocket or are involved in the kissing loop interaction between L2 & L3; for instance nucleotides in J1-2 (U22 and A23), J2-3 (G46, U47, C49, C50, U51, A52 and C53), L2 (U34, G37 and G38) and L3 (C60, C61 and A65,) are universally conserved. Several of the conserved inter-helical nucleotides interact with the bound purine via hydrogen bonds. The N3 and N9H atoms of the bound purine form a pair of hydrogen bonds with the Watson Crick base edge of U51 of J2-3, with N9H also forming a hydrogen bond to O2 of U47 of J2-3 [19]. In addition, N7 atom of the bound purine forms a hydrogen bond with the 2′-OH of U22 of J1-2 [19].

### SAM-I Riboswitches

In the SAM-I riboswitch structure [20], two tertiary motifs are of importance; a kink-turn and a pseudoknot [33] (Figure 2). This pseudoknot formation has been shown to be important for ligand binding [49]. We observed that nucleotides highly conserved in our alignment are the components of both the kink-turn and pseudoknot. The kink-turn in SAM-I riboswitch conforms to the consensus sequence as reported by [33] with minor variations. Variations were observed in some of the consensus base pairs and in the trinucleotide bulge separating the canonical stem (C-stem) from the non-canonical stem (NC-stem). Tandem G.A (pairs 35-20 and 36-19) in the NC-stem are highly conserved and this was expected as they play an important role in the formation of the kink-turn structure [50]. The identity of the closing base-pair of the C stem (pair G22-C30) is highly conserved whereas the NC (pair N17-N38) stem is not conserved with both U-A and C-G were encountered frequently (Figure 2). This indicates that any of the Watson-Crick pairs could form the flanking base pairs as long as base pairing is maintained. Furthermore, the trinucleotide bulge (residues 32, 33, and 34) also does not match the consensus (RNN) as uracil (U32) can replace R in the consensus. An A-minor interaction is present within the kink-turn structure where A36 in the NC-stem interacts with the G21-C31 pair in C-stem (Figure 2). This interaction is important in stabilizing the kinked structure [33] and was observed to be conserved in our alignments. As for the pseudoknot, the last two bases of hairpin loop P2 (G27 & G28) and first two bases of J3-4 (C65 & C66) are highly conserved whereas the other four bases involved in the pseudoknot formation are variable, consistent with the secondary structure diagram description [51].

We have identified an AGC amino-N3, N1-amino; Watson-Crick triple in which base A62 of J3-4 interacts with G23-C29 base pair in the P2b helix. This triple has been previously suggested to further tie J3-4 to helix P2b thereby stabilizing the pseudoknot formation [20]. Both the kink-turn and pseudoknot structures results in a binding pocket for SAM that sits in the minor grooves of P1 and P3. This in turn allows different faces of SAM to form hydrogen-bonding interactions to helix P3 and Van-der-Waals contacts to helix P1 [52]. Highly conserved nucleotides in the SAM-I riboswitch are found in helix P1 (U4, A6, U7, A87 and U88), junction J1/2 (G11, A12), helix P2a (G19, A20, G35, and A36), helix P2b (G21, A24, C31), helix P3 (G43, C44, A45, C47, G56, U57, G58 and C59) and J3/4 (A61). Many of these nucleotides constitute the tertiary interactions described previously. Three conserved nucleotides involving G11, C44 and G58 were previously reported to form a base triple which recognizes the main chain atoms of the methionine moiety [20]. Upon closer inspection, we found this interaction to be a type II G-minor interaction in which the guanosine docks the minor groove of a C-G Watson Crick receptor base pair instead of an adenosine. Additionally, two of the G-minor nucleotides (G11 and C44) are also part of a canonical ribose zipper interaction involving A12 of J1-2 and G43 of helix P3. This ribose zipper interaction appears to be highly conserved given the participation of G11 and C44 in recognizing the methionine tail of SAM [20]. Another important conserved nucleotide is U7 as its O2 carbonyl oxygen is involved in electrostatic interaction with the sulfur atom of SAM [20] that allows sensing of positively charged SAM functional group rather than depending on the negatively charged anionic phosphate backbone. Apart from these, several nucleotides in helix P3 such as the stacked G-C pairs (G56-C47, G58-C44 and G43-C59) were observed to have high sequence conservation. The conservation of these nucleotides is likely to be essential to assist the formation of stacking interactions involving the ligand and thereby stabilize the binding of SAM to the riboswitch.

### SAM-II Riboswitches

The SAM-II riboswitch folds into an H-type pseudoknot [13], a tertiary motif commonly adopted by viral RNAs that promote frameshifting during translation [53] (Figure 3). The H-type pseudoknot is characterized by the formation of two stem-loop structures where the loop regions (L1 and L2) interact with the paired regions (P2 and P1) to form major and minor groove triplexes respectively. A total of three base triples (G8-G42-C23, U11-A45-U21, and U12-A46-U20) were identified in the major groove triplex (formed by nucleotides of L1 and P2b) of SAM-II riboswitch where this triplex serves as the SAM-binding pocket. On closer visual examination, the SAM-binding pocket consists of five base triples, two of which were not annotated by NASSAM as they did not fit the search criteria. Two of the nucleotides in the U11-A45-U21 base triple have been shown to make direct contacts with SAM. Both the carbonyl oxygen’s (O4) of U11 & U21 are important for the recognition of the positively charged sulfur moiety and the methyl group of SAM [13], [52]. The base triples in the minor groove triplex form non-planar interactions where bases in L3 interact with two successive minor-groove base pairs in P1 via hydrogen bonds. We found two type II A-minor interactions in which A36 and A37 of L3 interacts with C4-G28 and G5-C27 pairs in helix P1 respectively. These interactions are part of the A-minor twist motif [13] previously reported in this structure. Additionally we also observed a CG/AA ribose zipper (C27-G28/A36-A37) interaction occurring within the A-minor twist motif. The extensive hydrogen bonding network provided by the combination of A-minor and ribose zipper interactions in addition to the base triples help to further anchor L3 to the minor groove of helix P1 and thereby stabilize the loop (L3) - helix (P1) interaction.

Nucleotides in loop L1 (U10, U11 and U12) and helix P2b (U20, U21, G22, C23, G42, C43, U44, A45, A46 and A47) were observed to be completely conserved as they are involved in SAM binding pocket formation [13]. Mutations in the P2 pseudoknot stem have been shown to prevent SAM recognition by the riboswitch thus implicating the importance of this stem formation for ligand binding [54]. All the major groove triples were observed to be highly conserved in the sequences aligned. Apart from U11 and U21, other key nucleotides in the base triples that were found to be conserved include U44 which is involved in hydrogen bonding interactions with the adenine moiety of SAM, A47 which interacts with both the amino and carboxylate groups of the methionine residue of SAM, and G22 that interacts with the 2′-OH group of adenine moiety of SAM [13]. The nucleotides in the minor groove triplex region were also highly conserved. Two nucleotides (G28 & A37) in the ribose-zipper interaction were observed not to be conserved where adenine occurs predominantly in position N28 while position N36 is variable with cytosine forming around 50% of the nucleotides found. It is also noted that these two nucleotides are part of the A-minor interaction (A36-C4-G28) occurring in the minor groove triplex. Further observation reveals uracil to be the predominant nucleotide in position N4 making the U4-A28 pairing to be highly conserved. The lack of adenine in position A36 is likely to prevent the formation of a functional A-minor interaction and therefore might be replaced with an alternative base-triple interaction.

### SAM-III Riboswitches

The SAM-III riboswitch folds into an inverted Y-shaped molecule [21] (Figure 4) and is one of the smallest riboswitches annotated in this study. Two highly conserved base-triples were found in this riboswitch. The first triple (which is an AGC triple) is formed by A73 (of J2-4) interacting with the Watson-Crick G90-C25 pair in helix P1. This base triple interaction occurs at the floor of the ligand binding pocket where it ties J2-4 to P1 SD-ASD helix and at the same time, helps to position N6 amine of A73 for SAM recognition [21]. Mutational studies had shown that changes to C25-G90 pairing results in decreased SAM binding activity although formation of a U25-G90 wobble can be tolerated albeit with a reduced binding affinity [21]. The second identified triple (A27-G66-G71) forms the ceiling of the ligand binding pocket together with the U72-A64 base pair. In this triple, G71 interacts with the Hoogsten faces of both A27 and G66 via hydrogen bonds resulting in P2 and J3-2 being weaved together.

A large number of nucleotides were observed to be conserved in this riboswitch where they are found in P1 (U22, C23, C24, G90, G91, G92, A93), P2 (G26, A27, G30, G31, C67, C68, U69, U70, G71, U72), J2-4 (A73, A74) and P4 (C75, C76, G88, G89). Generally these conserved nucleotides can be divided into two groups; first group include nucleotides involved in binding pocket formation (nucleotides in P2 and J2/4) and the second group include those that take part in the SD-ASD helix formation (nucleotides in P1 and P4). Nucleotides in P2 and J2-4 that are involved in crucial interactions with the ligand include G26 which make minor groove contacts with Watson-Crick face of SAM, A73 which form hydrogen bond interaction with the adenosyl moiety of SAM and both G71 and U72 which make electrostatic interactions with the positively charged sulfonium ion of SAM [21]. Conservation of nucleotides in P1 & P4 is crucial for the formation of the SD-ASD helix as the SD-ASD pairing is required for SAM-binding [21]. This in turn allows ligand binding and translational inhibition to occur simultaneously by the sequestration of the SD sequence.

### PreQ1 Riboswitches

Sequence alignments of preQ1 riboswitches [22] revealed a number of conserved nucleotides, most of which are found in the loop L3 region that is adenine rich (Figure 5). As with other H-type pseudoknots [53], L3 adenines are involved in base triple interactions with the minor groove of P1. Several other nucleotides that have been previously identified to be functional are highly conserved as well. These include G5, C17 and A30 that are all involved in hydrogen bonding interactions with the ligand [22], [55].

In this riboswitch, the ligand is sandwiched in between two identified AGC amino-N3, N1-amino; Watson-Crick triples (G11-A16-C31 & G5-C18-A28) which in turn maintains continuous stacking of the two helices (P1 & P2) (Figure 5). Both these triples show varying degrees of conservation in the sequence alignment. The G5-C18-A28 triple is conserved whereas the G11-A16-C31 triple is not. Base G5 of the first triple is involved in a hydrogen bonding interaction with the aminomethyl group of preQ1 via its O6 atom [22]. The second AGC triple is formed by A16 of loop L2 interacting with the minor groove of G11-C31 pair at the base of helix P2. Two base positions in the G11-A16-31 triple are variables as adenine can be substituted for guanine (G11) and accordingly, uracil can be substituted for guanine (A16). Interestingly, both these substitutions appear to occur together as a substitution of the position G11 to adenine will also see position A16 to change to uracil and thus, leading to the formation of a possible alternative base-triple interaction (A11-U16-C31). We also noted that this observation does not apply to all the sequences in our alignment such as in *Staphylococcus epidermidis* and *S. saprophyticus* where the presence of adenine in position 11 did not result in uracil to be present at position 16. At present there are no available structures that show the atomic details for this alternate triple. G11 is also involved in hydrogen bonding interaction with the aminomethyl group of preQ1 but via its pro-R_P_ phosphate oxygen [22]. Therefore, the formation of an alternative base triple may still accommodate this hydrogen bond interaction since this interaction lacks specificity as it does not require the edges of the base for the formation of the hydrogen bond [22].

A pseudo-cis ribose zipper was previously annotated in the *Thermoanaerobacter tencongensis* preQ1 riboswitch described in [42] and in this motif, A23 of L3 interacts with the A19-U2 pair in helix P1 [PDB: 3gca] where it facilitates a sharp bend that joins the 3′-P1 helix to the A-rich segment of loop L3. Although this interaction was previously annotated as a pseudo-cis ribose zipper interaction, this interaction can be considered to be a Type I A-minor interaction since A23 inserts itself into the minor groove of the A-U pair (Figure S11). Our structural alignment shows that this A-minor interaction is replaced by a base-triple interaction (G2-C21-A25) in the *Bacillus subtilis* preQ1 riboswitch [PDB: 3fu2] (Figure S11). In the *B. subtilis* preQ1 riboswitch, the A-U pair is replaced by a Watson-Crick C21-G2 pair and A25 utilizes its Watson Crick face to form hydrogen bonds to the sugar face of C21. Furthermore our alignment shows that adenine at sequence position 25 (numbering based on *B. subtilis* preQ1 riboswitch/PDB: 3fu2) to be fully conserved while the receptor base-pair (N21-N2) is variable, although we did observe the frequent occurrences of Watson-Crick base pairs. The presence of conserved adenine (A25) has the potential to enable other preQ1 riboswitches to utilize a similar A-minor interaction or other base-triple variants to perform the same function.

### Lysine Riboswitches

The five-way helix junction in the lysine riboswitch is stabilized by three important tertiary interactions: i) a kink-turn motif in P2, ii) a kissing-loop interaction formed between the terminal loops of P2 and P3 and iii) a loop (L4)-helix (P2) interaction [23] (Figure 6). The kink-turn interaction has been described in detail [56] where it helps to direct terminal loops L2 and L3 for the formation of a functional kissing-loop interaction. The formation of the kissing loop complex is essential for the biological function of the lysine riboswitch [56] and mutational studies have shown that any alterations to this loop-loop interaction leads to reduced transcription termination efficiency [56]. In the lysine riboswitch structure solved by [23], this kissing interaction is formed between residues 44-49 of L2 and residue 95-100 of L3. The sequence alignment shows that these six base pairs are poorly conserved among the aligned lysine riboswitch sequences. Presence of diverse nucleotides within the loop-loop interaction region probably enables the lysine riboswitch to adopt different kissing loop conformations to achieve the intended function.

The loop (L4)-helix (P2) interaction involves residues of pentaloop L4 and residues present in helix P2. This interaction is crucial for the organization of the ligand-binding site and for the riboswitch transcription attenuation control [57]. A conserved GAIM/AGN3 triple has been identified in which A127 of loop L4 interacts with the A23-G69 pair in the P2 helix. In addition, another base triple is formed between the highly conserved A126 of L4 and U24-G68 of P2. Mutations to the highly conserved adenines (A126 and A127) have been shown to cause a reduction in premature termination which reflects the importance of these nucleotides to the L4-P2 tertiary interaction as well as the reason behind the high sequence conservation of these nucleotides [57]. Together, these triples help to anchor parallel stems P2 and P4. This interaction can be considered to be a variant of the commonly encountered tetraloop-receptor interaction [35] because it is formed by a pentaloop and the recognition of the receptor helix is mediated by base triples rather than by the combination of A-minor and ribose zipper interactions. Additional tertiary interactions in the form of A-minor and base-quadruples were also encountered. The first A-minor is a conserved interaction formed between A81 of P3 and G14-C78 pair in P2 and therefore probably assists parallel arrangement of helices P2 and P3. A non-conserved A-minor interaction was found in the P2a-L2 turn formed by residues A51, G54 and C41. Upon closer inspection, we found this A-minor interaction to be part of a base quadruple interaction where C41 also forms a non-canonical interaction with A39. In the *T. maritima* lysine riboswitch, the P2a-L2 turn replaces the kink-turn motif found in other lysine riboswitch sequences [23]. As such this base quadruple interaction probably helps to stabilize the P2a-L2 turn, which in turn contributes to the proper alignment of hairpin loops L2 and L3 for the formation of the kissing-loop interaction (Figure 6). Another base triple was found between G163 of P1 and G141-A162 pair in P5. This base triple interaction is actually part of a purine quartet as G163 can also base pair with G11. The G11-G163 base pair is situated in the lower layer of nucleotides involved in the ligand binding pocket formation and thus, this purine quartet forms the floor of the ligand-binding pocket and thereby helps to stabilize top of helix P1 that is important for controlling gene expression [23].

Comparative sequence alignment of lysine riboswitches revealed highly conserved nucleotides in helix P1 (G9, A10, G11, G163, and C166), helix P2 (G12, G14, C15, A23, U28, G69, G77, C78 and C79), J2-3 (G80, A81), helix P4 (G114, U140), L4 (A126 and A127) and helix P5 (G141). Most of these nucleotides involve terminal residues of the helices that make up the binding pocket, completely encapsulating lysine and therefore accounting for the high conservation of these residues. Nucleotides involved in the long-distance tertiary interactions were found to be highly conserved as well. It is likely that these interactions are crucial for the proper arrangement of the helices that in turn allows the terminal residues of the helices to be aligned in a proper orientation to form the lysine binding pocket.

### FMN Riboswitches

The complex FMN-bound junctional site is linked together by two almost identical domains P2-P6 and P3-P5 (Figure 7) [24]. Each domain has two stem-loop structures, stabilized by two pairs of tertiary interactions involving loop-loop (L2-L6 and L3-L5) and loop-helix (L6-P2 and L3-P5) interactions. The loop-loop interactions in the FMN riboswitch do not conform to the commonly observed kissing-loop interaction found in other RNAs [34], [58]. The loop-loop pairs (L2-L6 and L3-L5) have no complementary sequence to each other. Both L2 and L5 have a T-loop like conformation with the sequence UGAAA (Figure 7). The T-loop motif has been previously observed in ribosomes and ribozymes where it is involved in various tertiary interactions [59], [60]. The large gap present between the fourth and fifth nucleotides in the motif is capable of accepting an intercalating base. In this riboswitch, A90 of L6 intercalates between A21 and A22 of L2 and A38 of L3 intercalates between A72 and A73 of L5, thus linking the loop-loop (L2-L6 and L3-L5) regions together.

A number of other tertiary interactions including A-minor motifs and a base triple were also found. A total of four A/G-minor motifs (A63-C82-G41, A104-C46-G33, G11-G84-C31, and G62-G32-C83) (Figure 7) were found in our annotation. Most of the nucleotides involved in the A/G-minor interactions are found in interhelical regions J2-3 and J5-6. G11-G84-C31 links bases in J1-2, J2-3 and J5-6 while G62-G32-C83 links bases in J4-5, J2-3 and J5-6. In addition, a few of the nucleotides in the A/G-minor motifs have been shown to be involved in the recognition of the ligand [24]. The G11, G32 and G84 are involved in hydrogen bonding interactions with the phosphate oxygens whereas G33 and G41 interact with the phosphate oxygens via Mg^2+^ mediated water molecules. As such the A/G-minor motifs appear to be involved in the recognition of FMN’s phosphate oxygens in addition to linking the two domains together. A GCG triple was identified whereby G12 of J1-2 interacts with the C30 (J2-3)-G93 (L6) pair. In order to prevent a steric clash, G12 is positioned in a non-planar orientation resulting in both G12-G93-C30 and G11-G84-C31 interactions to be weakened. It has been hypothesized that weakening of both these interactions could enhance the mobility of J1-2 segment and therefore influence gene regulation as this segment is part of the switching helix P1 involved in the stabilization/destabilization of the terminator stem [24].

A large number of nucleotides are highly conserved in this riboswitch because they are involved in the various tertiary interactions described previously. The L2 T-loop was commonly observed having the UGAAA sequence whereas the L5 T-loop sequence was observed to be more flexible since the second adenine (A72) in this motif is less conserved when compared with the equivalent position (A21) in the L2 T-loop. The G12-G93-C30 base triple interaction was also found to be highly conserved. Position C30 of the triple is not conserved because uracil occurs occasionally in this position. Furthermore, all the nucleotides involved in the A-minor interactions were observed to be highly conserved indicating that the A/G-minor interactions play significant roles in the overall structure of the riboswitch.

### TPP Riboswitches

Both the *Arabidopsis thaliana* [PDB: 3d2v] [43] and *Escherichia coli* [PDB: 2gdi] [25] TPP riboswitches superimposes well with a RMSD value of 0.8 (Figure 8). In the *E. coli* TPP riboswitch, two important tertiary interactions are present which help to bring together the pyrimidine and pyrophosphate sensor helices and eventually helps to stabilize the global fold of the riboswitch [61]. The first involves a loop-loop receptor interaction where loop L5 (U68, A69, A70 and U71) interacts with residues present in helix P3. As with other classic loop-loop receptor interactions, the loop residues are involved in both A-minor and ribose zipper interactions with residues in the receptor helix. In this manner, both A70 and U71 of L5 are involved in a ribose zipper interaction with G21 and C22 of P3 while A70 is also involved in an A-minor interaction with the C22-G37 pair. The second tertiary interaction involves formation of a pair stacked tetrads between the non-canonical base pairs A56-G83 and A53-A84 and the minor-groove of G17-C49 and G16-C50 present in helix P2. The base-quadruples can be further broken down into two separate A-minor interactions. Both A56 and A84 are involved in type I A-minor interactions with G17-C49 and G16-C50 pairs respectively. The base quadruples help to stabilize the J2-4 junction and allows the pyrimidine and pyrophosphate helices to be aligned in parallel.

In addition, two different triples were identified in which the first triple, a GGA triple (G19-G42-A47; Numbering based on 2gdi), was identified in the *E. coli* TPP riboswitch whereas the second triple, an AUA triple (A43-A68-U47: Numbering based on 3d2v), was identified in the *A. thaliana* TPP riboswitch. The G19-G42-A47 triple links bases in the highly conserved J3-2 T-loop and helix P2. An additional tertiary interaction is present in this region where A41 is involved in a highly conserved Type II A-minor interaction with G18-C48 pair. The G19-G42-A47 triple stacks on top A41-G18-C48 A-minor interaction and together, these interactions help to stabilize the binding of HMP ring of TPP to the pyrimidine sensor helix [61]. As for the AUA triple in eukaryotic TPP riboswitch, it is found to bridge helix P4 and junction J2-4. Helix P4 is part of the stacked helices required for the recognition of the pyrophosphate group [61]. As such, this AUA triple has a high probability of stabilizing the interaction between the pyrophosphate sensor helix and the pyrophosphate moiety of TPP.

We observed that most of the tertiary interactions are structurally conserved in both *A. thaliana* and *E. coli* TPP riboswitches albeit with minor variations. For the A53-A84-G16-C50 quadruple, guanine replaces A53 in the *A. thaliana* riboswitch (G42-A72-G8-C38). Similarly for the G19-G42-A47 triple, guanine replaces A47 in the *A. thaliana* riboswitch (G11-G30-G34). Presence of guanine alters the hydrogen-bonding pattern in both these interactions in *A. thaliana* riboswitch (Figure S12). As an example, in the triple interaction, G34 uses its Hoogsteen face instead of Watson-Crick face used by A47 in the prokaryotic TPP riboswitch to form hydrogen bonds to Watson-Crick face of G11 and additionally, to sugar face of G30 (Figure S12). The base in position 47 of the triple is not conserved, although we did notice adenine to occur more frequently at this position. The second base triple (A43-A68-U47) annotated in the *A. thaliana* riboswitch was not found in the *E. coli* riboswitch structure (C55-A80-U59) although the sequence for this triple is highly conserved in other prokaryotic TPP riboswitch sequences (Figure 8). Therefore the *E. coli* riboswitch might serve as an exception with this triple replaced by an alternative structural interaction. In addition, all four A-minor motifs were found to be structurally conserved in *A. thaliana* and *E. coli* TPP riboswitches.

Highly conserved nucleotides in this riboswitch are found in the functionally important regions of the riboswitch namely in helix J3-2 (U39, G40, A41, G42, A43), P4 (U59, A80), J4-5 (G60), and J5-4 (C77, G78). Many of the highly conserved nucleotides participate in the formation of key structural interactions such as the J3-2 T-loop motif which is formed by residues 39–43. This interaction is important for the recognition of the pyrimidine moiety of TPP [61]. Residues 59–60 and 77–78 are situated close to the binding of pyrophosphate moiety where G60, C77 and G78 have been shown to interact with one of the two manganese ions (M_A_) that take part in the coordination of pyrophosphate group whereas G78 is the only residue among them that makes a direct contact to the pyrophosphate group [61].

### Magnesium Riboswitches

The majority of tertiary interactions in the Mg^2+^ riboswitches or previously known as the ykoK leader [62] are found in P2-L5-L4 regions since these regions are located near to the Mg^2+^ binding sites and anti-terminator nucleotides (Figure 9) [26]. Five A-minor motifs were observed in our annotation where four (A88-G151-C33, A117-G83-C57, A71-G22-C163 and A155-G107-C99) of them have been previously described in the literature where they help to bring the parallel helices into close contact [63]. The additional A-minor interaction found in this annotation is formed between A105 of L5 and G73-C68 pair at the base of the P4 helix. A105 is also involved in a non-canonical pairing with A72 of L4 and thus, this interaction helps to link both L4 and L5. Additional long-range tertiary interactions involving a tetraloop-tetraloop receptor and base triples are also present in this riboswitch. In the tetraloop-tetraloop receptor interaction, the CAAA (residues 69-72) loop of L4 docks to the base of P2 helix via A-minor and ribose zipper interactions. Both A70 and A71 are involved in a ribose zipper interaction with C163 and G164 while A71 forms a type II A-minor interaction with G22-C163 pair. Interestingly this CAAA tetraloop has a GNRA like conformation. Our alignment shows that the CAAA sequence is not conserved as other Mg^2+^ riboswitches adopt GNRA sequences at this position with both GAAA and GAGA commonly encountered.

Furthermore, three different base triples were annotated in this riboswitch where all three triples seem to be involved in different roles. In the first triple, U24, which is a bulged-out residue in helix P2, interacts with the G100-A106 pair in helix P5. This interaction helps to further anchor P5 to P2 together with the help of A155-G107-C99 A-minor motif. As expected, this triple was highly conserved since all three bases of the triple are located near the Mg^2+^ binding sites and might directly interact with the metals. The second triple (A46-U138-A139) is formed by the junctional residues of J2-3 and J6-2 and is situated between the base of helix P6 and the apex of helix P2. This base triple appears to be involved in maintaining a continuous base stacking between the bases of P2 and P6. In the final base triple, C35, which is a bulged-out residue in P2, interacts with the U34-U150 pair below it. This U-U pair sits on top of an A-minor C-G receptor base-pair (C33-G151) which is responsible for weaving J4-5 interhelical region with helix P2. Therefore, this triple probably serves to stabilize the bulged-out residue or helps to stabilize the nearby A-minor interaction.

A total of twenty-six nucleotides were found to be highly conserved in this riboswitch where they are largely distributed in the P2-L5-L4 regions. Nucleotides in these regions are located near the Mg^2+^ binding sites and assist in coordinating the P2-L5-L4 network of tertiary interactions. As an example, residues G22, U24, C99 and C163 are located near the Mg1 and Mg2 binding sites and U24 also forms a base-triple with a Watson-Crick base pair at the base of helix P5 which helps to anchor L5 to P2. All the tertiary interactions were observed to be highly conserved except the C35-U34-U150 triple. Our alignments show that the identity of the bulged-out residue at position 35 is variable with cytosine forming 60% of the nucleotides found in this position while U34-U150 pairing forms the dominant receptor base-pair. We noticed that the sequence of the receptor base-pair seems to change with the identity of residue 35. For example, if C is present at sequence position 35, the receptor base pair was observed to be an U-U but when A is present, U-A would form the receptor base pair.

### c-di-GMP Riboswitches

The most notable tertiary interaction in the c-di-GMP riboswitch is a tetraloop-receptor interaction formed between terminal loop of P2 (residues 32-35) with receptor helix P3 (Figure 10) [27]. The terminal loop belongs to the GNRA (GAAA) fold where both A33 and A34 are involved in a ribose zipper interaction with C59 and U60 in helix P3. Both A34 and A35 of the terminal loop are also involved in A-minor interactions with A78-U60 and G79-C59 pairs respectively. The A35-G79-C59 A-minor motif was found to be highly conserved whereas the other A-minor motif was observed not to be conserved. Guanine was observed to occur infrequently in the third position (sequence position 34) of the tetraloop. A previous study has shown that type I/IIP A-minor interactions, which are characteristic of GNRA/receptor interactions, can tolerate guanine at the third GNRA position [44]. Therefore, other c-di-GMP riboswitches might use this G-minor interaction together with the A35-G79-U60 interaction to mediate the recognition process between the loop and receptor residues.

In addition, A49 of J2-3 is involved in a highly conserved Type-I A-minor like interaction with the G45-C22 pair in helix P2. This interaction assists the stacking interactions between A47 and the guanosine bases of c-di-GMP (Gα and Gβ). Furthermore, the G14-C93 pair at the apex of the P1 helix interacts with A95, a bulged-out residue in helix P1, to form an AGC base triple. The G14-C93 pair is also involved in the extensive stacking network involving the two bases of c-di-GMP and therefore, this triple is likely to enhance the stacking interactions which in turn help to stabilize helix P1. This triple is only moderately conserved as the identities of the residues at position 14 and 95 are considered as variables. However, we did observe guanine (N14) and adenine (N95) to occur more frequently at those respective positions.

Highly conserved nucleotides in this riboswitch are found in J1-2 (G20), P2 (C22, A23, A25, G32, A35, G42, C44 and G45), J2-3 (A47, A48, A49), P3 (C59, G79, G83 and G90) and J3-1 (C92). The interhelical nucleotides are important in this riboswitch because they form the ligand-binding pocket [27]. Key conserved interhelical nucleotides required for the recognition of the ligand are G20, C92, A47 and A48 [27]. A conserved pairing was observed involving C44, which is part of an internal loop in helix P2 and G83, which is a bulged-out residue in helix P3. This interaction is important in mediating the parallel arrangement of helices P2 and P3.

## Discussion

The tertiary motifs found in riboswitches can be generally divided into two groups; first group include tertiary motifs which are involved in the formation of ligand-binding pockets or recognize the functional groups in a ligand whereas the second group consist of tertiary motifs which are involved in long-distance tertiary interactions.

Base-triples being one of the most common tertiary interactions encountered in riboswitches play three major roles namely; form ligand-binding pockets, constituents of more complex structural interactions and are involved in RNA helix packing. More than half of the triples annotated in this study are involved in the formation of ligand binding pockets. These triples help to stabilize RNA-ligand interactions and at the same time, few of them are also involved in the recognition of the ligand. Ligand-binding pockets which consist of stacks of base-triples have been reported before in artificially evolved aptamers such as FMN binding aptamer [64], theophylline aptamer [65], malachite aptamer [66] and also in the Group I *Tetrahymena* ribozyme [67]. This strategy is utilized by RNA to form a tight binding pocket for the recognized substrate. Base-triples involved in the formation of ligand binding-pockets were observed to be highly conserved. Other tertiary motifs that are involved in the recognition of the ligand’s functional groups such as the ribose-zipper interaction in the SAM-I riboswitch and also the G-minors in the FMN riboswitch were also observed to be highly conserved. This was expected as extreme changes to the sequences of these interactions might lead to the formation of non-functional motifs and in turn alter the ligand recognition process.

The tertiary motifs in the second group can be further divided into two groups: i) simple tertiary motifs like base-triples and A-minor interactions and ii) complex tertiary motifs such as loop-receptor interactions and kink-turns. These motifs have varying levels of conservation although the majority of them are highly conserved. One particular tertiary motif that is highly conserved in riboswitches is the A-minor motif. A-minor interactions are highly conserved in the ribosomal subunits where they help the ribosome to form a densely compact structure [30]. Similarly in riboswitches, these interactions help to link distant helices in the riboswitch structures, which in turn allow the different helices to be packed together to form a compact structure. It is interesting to note about the presence of variant A-minor motifs such as G-minors in our annotation. Interestingly, all three G-minors found appear to be involved in crucial functional roles and were observed to be highly conserved in the sequence alignments. We anticipated that the type-II G-minor motif might be more widespread than previously hypothesized and correspondingly, a more comprehensive annotation of available RNA structures is likely to uncover the existences of other A-minor variants such as those discussed by Xin et al [68].

The ribose-zipper motifs were found to be sparsely conserved although several of the ribose-zipper sequences had highly specific sequence patterns (e.g., preferences for purines at the loop-side residues). Previously, several types of ribose-zippers have been found in specific sequence contexts [32], [69] in which the formation of these interactions may be attributed to the surrounding structural elements [70]. For riboswitches, the loop-side residues of the ribose-zipper motif are usually involved in specific minor-groove contacts (such as forming the A-minor interaction), which is the primary reason behind the sequence specificity of this motif.Complex tertiary motifs were observed to be highly conserved, however the nucleotides involved in their formation are not entirely conserved. These complex tertiary motifs can be broken down into component motifs such as adenosine platforms [71], sheared G-A base pairs [72], base-triples [29], [31] or A-minor motifs [30]. These interactions help to stabilize the formation of complex tertiary motifs [33] or are involved in important recognition process between different structural elements that form the tertiary motif [44]. For example, in a tetraloop-receptor interaction, the GNRA tetraloop is usually not conserved in which the loop can be formed by different combination of nucleotides such as GCAA, GGAG and GAAA. But the last two nucleotides in tetraloop are observed to be highly conserved, either A or G since these nucleotides form A/G-minor motifs which are involved in the recognition process between the tetraloop and the receptor helix.

Some tertiary interactions were found to be not conserved despite being perceived as possibly conserved due to their locations in the structures. Examples of these are the base-triples involving a bulged-out base in Mg^2+^ and c-di-GMP riboswitches. Since these interactions are not involved in any obvious functional roles, it can be assumed that these interactions are ‘loose’ and therefore, the sequences of these interactions might vary with different species. Previous studies have shown that base triples can be formed by different combination of bases while still retaining the same orientation and geometry [29], [31].

### Conclusions

Our survey reveals that conserved nucleotides in riboswitches are either mainly involved in the formation of ligand-binding pockets or alternatively participate in tertiary interactions that are most likely to be vital for the structure as well as the function of the riboswitches. In addition, some unusual tertiary interactions such as G-minors, a pentaloop-receptor interaction and loop-loop interactions involving non-complementary sequences were also found. This demonstrates that different riboswitches are able to utilize variants of known RNA tertiary motifs in order to achieve a similar function. Mutations of highly conserved tertiary interactions will be expected to destabilize the structures or to alter the normal function of riboswitches that in turn may lead to new strategies in the use of riboswitches as novel antibacterial targets.

## Supporting Information

Figure S1 **Rfam seed alignment for purine riboswitches.** Blue shaded columns represent nucleotide positions that are more than 95% conserved.
(PNG)

Figure S2 **Rfam seed alignment for SAM-I riboswitches.** Blue shaded columns represent nucleotide positions that are more than 95% conserved.
(PNG)

Figure S3 **Rfam seed alignment for SAM-II riboswitches.** Blue shaded columns represent nucleotide positions that are more than 95% conserved.
(PNG)

Figure S4 **Rfam seed alignment for SAM-III riboswitches.** Blue shaded columns represent nucleotide positions that are more than 95% conserved.
(PNG)

Figure S5 **Rfam seed alignment for preQ1 riboswitches.** Blue shaded columns represent nucleotide positions that are more than 95% conserved.
(PNG)

Figure S6 **Rfam seed alignment for lysine riboswitches.** Blue shaded columns represent nucleotide positions that are more than 95% conserved.
(PNG)

Figure S7 **Rfam seed alignment for FMN riboswitches.** Blue shaded columns represent nucleotide positions that are more than 95% conserved.
(PNG)

Figure S8 **Rfam seed alignment for TPP riboswitches.** Blue shaded columns represent nucleotide positions that are more than 95% conserved.
(PNG)

Figure S9 **Rfam seed alignment for Mg^2+^ riboswitches.** Blue shaded columns represent nucleotide positions that are more than 95% conserved.
(PNG)

Figure S10 **Rfam seed alignment for c-di-GMP riboswitches.** Blue shaded columns represent nucleotide positions that are more than 95% conserved.
(PNG)

Figure S11 **Pseudo-cis ribose zipper interaction previously annotated in the** ***T. tencongensis*** **preQ1 riboswitch.** A) A23-A19-U2 A-minor in *T. tencongensis* preQ1 riboswitch [PDB: 3gca]. B) A25-C21-G2 base-triple in *B. subtilis* preQ1 riboswitch [PDB: 3fu2].
(TIFF)

Figure S12 **Differences in hydrogen bonding patterns between equivalent base interactions in TPP riboswitches.** (A) G19-A42-A47 triple in *E. coli* TPP riboswitch [PDB: 2gdi] (B) G11-G30-G34 triple in *A. thaliana* riboswitch [PDB: 3d2v]. (C) A53-A84-G16-C50 quadruple in *E. coli* TPP riboswitch [PDB: 2gdi] (D) G42-A72-G8-C38 quadruple in *A. thaliana* TPP riboswitch [PDB: 3d2v].
(TIFF)

Table S1 **List of sequences and structures used for riboswitch sequence-structure comparisons.**
(DOC)

Table S2 **List of annotated tertiary motifs in riboswitches.**
(DOC)

Table S3 **List of highly conserved nucleotides in riboswitches.**
(DOC)

## References

[1] Mandal M, Breaker RR (2004) Gene regulation by riboswitches. Nat Rev Mol Cell Biol 5: 451–463. 15173824 10.1038/nrm1403

[2] Serganov A (2009) The long and the short of riboswitches. Curr Opin Struct Biol 19: 251–259. 19303767 10.1016/j.sbi.2009.02.002 PMC2762789

[3] Tucker BJ, Breaker RR (2005) Riboswitches as versatile gene control elements. Curr Opin Struct Biol 15: 342–348. 15919195 10.1016/j.sbi.2005.05.003

[4] Wachter A, Tunc-Ozdemir M, Grove BC, Green PJ, Shintani DK, et al (2007) Riboswitch control of gene expression in plants by splicing and alternative 3′ end processing of mRNAs. Plant Cell 19: 3437–3450. 17993623 10.1105/tpc.107.053645 PMC2174889

[5] Henkin TM (2008) Riboswitch RNAs: using RNA to sense cellular metabolism. Genes Dev 22: 3383–3390. 19141470 10.1101/gad.1747308 PMC3959987

[6] Winkler WC, Breaker RR (2005) Regulation of bacterial gene expression by riboswitches. Annu Rev Microbiol 59: 487–517. 16153177 10.1146/annurev.micro.59.030804.121336

[7] Winkler WC, Nahvi A, Roth A, Collins JA, Breaker RR (2004) Control of gene expression by a natural metabolite-responsive ribozyme. Nature 428: 281–286. 15029187 10.1038/nature02362

[8] Kwon M, Strobel SA (2008) Chemical basis of glycine riboswitch cooperativity. RNA 14: 25–34. 18042658 10.1261/rna.771608 PMC2151043

[9] Mandal M, Lee M, Barrick JE, Weinberg Z, Emilsson GM, et al (2004) A glycine-dependent riboswitch that uses cooperative binding to control gene expression. Science 306: 275–279. 15472076 10.1126/science.1100829

[10] Garst AD, Batey RT (2009) A switch in time: detailing the life of a riboswitch. Biochim Biophys Acta 1789: 584–591. 19595806 10.1016/j.bbagrm.2009.06.004 PMC2783387

[11] Wakeman CA, Winkler WC, Dann CE (2007) Structural features of metabolite-sensing riboswitches. Trends Biochem Sci 32: 415–424. 17764952 10.1016/j.tibs.2007.08.005 PMC2933830

[12] Gilbert SD, Love CE, Edwards AL, Batey RT (2007) Mutational analysis of the purine riboswitch aptamer domain. Biochemistry 46: 13297–13309. 17960911 10.1021/bi700410g PMC2556308

[13] Gilbert SD, Rambo RP, Van Tyne D, Batey RT (2008) Structure of the SAM-II riboswitch bound to S-adenosylmethionine. Nat Struct Mol Biol 15: 177–182. 18204466 10.1038/nsmb.1371

[14] Calnan BJ, Tidor B, Biancalana S, Hudson D, Frankel AD (1991) Arginine-mediated RNA recognition: the arginine fork. Science 252: 1167–1171. 1709522 10.1126/science.252.5009.1167

[15] Brodsky AS, Williamson JR (1997) Solution structure of the HIV-2 TAR-argininamide complex. J Mol Biol 267: 624–639. 9126842 10.1006/jmbi.1996.0879

[16] Ye X, Kumar RA, Patel DJ (1995) Molecular recognition in the bovine immunodeficiency virus Tat peptide-TAR RNA complex. Chem Biol 2: 827–840. 8807816 10.1016/1074-5521(95)90089-6

[17] Ye X, Gorin A, Ellington AD, Patel DJ (1996) Deep penetration of an alpha-helix into a widened RNA major groove in the HIV-1 rev peptide-RNA aptamer complex. Nat Struct Biol 3: 1026–1033. 8946856 10.1038/nsb1296-1026

[18] Berman HM, Westbrook J, Feng Z, Gilliland G, Bhat TN, et al (2000) The Protein Data Bank. Nucleic Acids Res 28: 235–242. 10592235 10.1093/nar/28.1.235 PMC102472

[19] Serganov A, Yuan YR, Pikovskaya O, Polonskaia A, Malinina L, et al (2004) Structural basis for discriminative regulation of gene expression by adenine- and guanine-sensing mRNAs. Chem Biol 11: 1729–1741. 15610857 10.1016/j.chembiol.2004.11.018 PMC4692365

[20] Montange RK, Batey RT (2006) Structure of the S-adenosylmethionine riboswitch regulatory mRNA element. Nature 441: 1172–1175. 16810258 10.1038/nature04819

[21] Lu C, Smith AM, Fuchs RT, Ding F, Rajashankar K, et al (2008) Crystal structures of the SAM-III/S(MK) riboswitch reveal the SAM-dependent translation inhibition mechanism. Nat Struct Mol Biol 15: 1076–1083. 18806797 10.1038/nsmb.1494 PMC3467307

[22] Klein DJ, Edwards TE, Ferre-D’Amare AR (2009) Cocrystal structure of a class I preQ1 riboswitch reveals a pseudoknot recognizing an essential hypermodified nucleobase. Nat Struct Mol Biol 16: 343–344. 19234468 10.1038/nsmb.1563 PMC2657927

[23] Serganov A, Huang L, Patel DJ (2008) Structural insights into amino acid binding and gene control by a lysine riboswitch. Nature 455: 1263–1267. 18784651 10.1038/nature07326 PMC3726722

[24] Serganov A, Huang L, Patel DJ (2009) Coenzyme recognition and gene regulation by a flavin mononucleotide riboswitch. Nature 458: 233–237. 19169240 10.1038/nature07642 PMC3726715

[25] Serganov A, Polonskaia A, Phan AT, Breaker RR, Patel DJ (2006) Structural basis for gene regulation by a thiamine pyrophosphate-sensing riboswitch. Nature 441: 1167–1171. 16728979 10.1038/nature04740 PMC4689313

[26] Ramesh A, Wakeman CA, Winkler WC (2011) Insights into metalloregulation by M-box riboswitch RNAs via structural analysis of manganese-bound complexes. J Mol Biol 407: 556–570. 21315082 10.1016/j.jmb.2011.01.049 PMC3056551

[27] Smith KD, Lipchock SV, Livingston AL, Shanahan CA, Strobel SA (2010) Structural and biochemical determinants of ligand binding by the c-di-GMP riboswitch. Biochemistry 49: 7351–7359. 20690679 10.1021/bi100671e PMC3146058

[28] Hamdani HY, Appasamy SD, Willett P, Artymiuk PJ, Firdaus-Raih M (2012) NASSAM: a server to search for and annotate tertiary interactions and motifs in three-dimensional structures of complex RNA molecules. Nucleic Acids Res. 10.1093/nar/gks513 PMC3394293 22661578

[29] Firdaus-Raih M, Harrison AM, Willett P, Artymiuk PJ (2011) Novel base triples in RNA structures revealed by graph theoretical searching methods. BMC Bioinformatics 12 Suppl 13S2. 10.1186/1471-2105-12-S13-S2 PMC3278836 22373013

[30] Nissen P, Ippolito JA, Ban N, Moore PB, Steitz TA (2001) RNA tertiary interactions in the large ribosomal subunit: the A-minor motif. Proc Natl Acad Sci U S A 98: 4899–4903. 11296253 10.1073/pnas.081082398 PMC33135

[31] Abu Almakarem AS, Petrov AI, Stombaugh J, Zirbel CL, Leontis NB (2012) Comprehensive survey and geometric classification of base triples in RNA structures. Nucleic Acids Res 40: 1407–1423. 22053086 10.1093/nar/gkr810 PMC3287178

[32] Tamura M, Holbrook SR (2002) Sequence and structural conservation in RNA ribose zippers. J Mol Biol 320: 455–474. 12096903 10.1016/s0022-2836(02)00515-6

[33] Klein DJ, Schmeing TM, Moore PB, Steitz TA (2001) The kink-turn: a new RNA secondary structure motif. EMBO J 20: 4214–4221. 11483524 10.1093/emboj/20.15.4214 PMC149158

[34] Chang KY, Tinoco I Jr (1994) Characterization of a “kissing” hairpin complex derived from the human immunodeficiency virus genome. Proc Natl Acad Sci U S A 91: 8705–8709. 8078946 10.1073/pnas.91.18.8705 PMC44675

[35] Costa M, Michel F (1995) Frequent use of the same tertiary motif by self-folding RNAs. EMBO J 14: 1276–1285. 7720718 10.1002/j.1460-2075.1995.tb07111.x PMC398207

[36] Pleij CW, Rietveld K, Bosch L (1985) A new principle of RNA folding based on pseudoknotting. Nucleic Acids Res 13: 1717–1731. 4000943 10.1093/nar/13.5.1717 PMC341107

[37] Yang H, Jossinet F, Leontis N, Chen L, Westbrook J, et al (2003) Tools for the automatic identification and classification of RNA base pairs. Nucleic Acids Res 31: 3450–3460. 12824344 10.1093/nar/gkg529 PMC168936

[38] Gardner PP, Daub J, Tate JG, Nawrocki EP, Kolbe DL, et al (2009) Rfam: updates to the RNA families database. Nucleic Acids Res 37: D136–140. 18953034 10.1093/nar/gkn766 PMC2686503

[39] Nawrocki EP, Kolbe DL, Eddy SR (2009) Infernal 1.0: inference of RNA alignments. Bioinformatics 25: 1335–1337. 19307242 10.1093/bioinformatics/btp157 PMC2732312

[40] Waterhouse AM, Procter JB, Martin DM, Clamp M, Barton GJ (2009) Jalview Version 2–a multiple sequence alignment editor and analysis workbench. Bioinformatics 25: 1189–1191. 19151095 10.1093/bioinformatics/btp033 PMC2672624

[41] Pettersen EF, Goddard TD, Huang CC, Couch GS, Greenblatt DM, et al (2004) UCSF Chimera–a visualization system for exploratory research and analysis. J Comput Chem 25: 1605–1612. 15264254 10.1002/jcc.20084

[42] Spitale RC, Torelli AT, Krucinska J, Bandarian V, Wedekind JE (2009) The structural basis for recognition of the PreQ0 metabolite by an unusually small riboswitch aptamer domain. J Biol Chem 284: 11012–11016. 19261617 10.1074/jbc.C900024200 PMC2670106

[43] Thore S, Leibundgut M, Ban N (2006) Structure of the eukaryotic thiamine pyrophosphate riboswitch with its regulatory ligand. Science 312: 1208–1211. 16675665 10.1126/science.1128451

[44] Geary C, Baudrey S, Jaeger L (2008) Comprehensive features of natural and in vitro selected GNRA tetraloop-binding receptors. Nucleic Acids Res 36: 1138–1152. 18158305 10.1093/nar/gkm1048 PMC2275092

[45] Batey RT, Gilbert SD, Montange RK (2004) Structure of a natural guanine-responsive riboswitch complexed with the metabolite hypoxanthine. Nature 432: 411–415. 15549109 10.1038/nature03037

[46] Gilbert SD, Batey RT (2006) Riboswitches: fold and function. Chem Biol 13: 805–807. 16931328 10.1016/j.chembiol.2006.08.002

[47] Mulhbacher J, Lafontaine DA (2007) Ligand recognition determinants of guanine riboswitches. Nucleic Acids Res 35: 5568–5580. 17704135 10.1093/nar/gkm572 PMC2018637

[48] Mandal M, Boese B, Barrick JE, Winkler WC, Breaker RR (2003) Riboswitches control fundamental biochemical pathways in Bacillus subtilis and other bacteria. Cell 113: 577–586. 12787499 10.1016/s0092-8674(03)00391-x

[49] McDaniel BA, Grundy FJ, Henkin TM (2005) A tertiary structural element in S box leader RNAs is required for S-adenosylmethionine-directed transcription termination. Mol Microbiol 57: 1008–1021. 16091040 10.1111/j.1365-2958.2005.04740.x

[50] Moore T, Zhang Y, Fenley MO, Li H (2004) Molecular basis of box C/D RNA-protein interactions; cocrystal structure of archaeal L7Ae and a box C/D RNA. Structure 12: 807–818. 15130473 10.1016/j.str.2004.02.033

[51] Barrick JE, Breaker RR (2007) The distributions, mechanisms, and structures of metabolite-binding riboswitches. Genome Biol 8: R239. 17997835 10.1186/gb-2007-8-11-r239 PMC2258182

[52] Wang JX, Breaker RR (2008) Riboswitches that sense S-adenosylmethionine and S-adenosylhomocysteine. Biochem Cell Biol 86: 157–168. 18443629 10.1139/O08-008

[53] Staple DW, Butcher SE (2005) Pseudoknots: RNA structures with diverse functions. PLoS Biol 3: e213. 15941360 10.1371/journal.pbio.0030213 PMC1149493

[54] Corbino KA, Barrick JE, Lim J, Welz R, Tucker BJ, et al (2005) Evidence for a second class of S-adenosylmethionine riboswitches and other regulatory RNA motifs in alpha-proteobacteria. Genome Biol 6: R70. 16086852 10.1186/gb-2005-6-8-r70 PMC1273637

[55] Roth A, Winkler WC, Regulski EE, Lee BW, Lim J, et al (2007) A riboswitch selective for the queuosine precursor preQ1 contains an unusually small aptamer domain. Nat Struct Mol Biol 14: 308–317. 17384645 10.1038/nsmb1224

[56] Blouin S, Lafontaine DA (2007) A loop loop interaction and a K-turn motif located in the lysine aptamer domain are important for the riboswitch gene regulation control. RNA 13: 1256–1267. 17585050 10.1261/rna.560307 PMC1924893

[57] Blouin S, Chinnappan R, Lafontaine DA (2011) Folding of the lysine riboswitch: importance of peripheral elements for transcriptional regulation. Nucleic Acids Res 39: 3373–3387. 21169337 10.1093/nar/gkq1247 PMC3082890

[58] Friebe P, Boudet J, Simorre JP, Bartenschlager R (2005) Kissing-loop interaction in the 3′ end of the hepatitis C virus genome essential for RNA replication. J Virol 79: 380–392. 15596831 10.1128/JVI.79.1.380-392.2005 PMC538730

[59] Krasilnikov AS, Mondragon A (2003) On the occurrence of the T-loop RNA folding motif in large RNA molecules. RNA 9: 640–643. 12756321 10.1261/rna.2202703 PMC1370430

[60] Nagaswamy U, Fox GE (2002) Frequent occurrence of the T-loop RNA folding motif in ribosomal RNAs. RNA 8: 1112–1119. 12358430 10.1017/s135583820202006x PMC1370325

[61] Miranda-Rios J (2007) The THI-box riboswitch, or how RNA binds thiamin pyrophosphate. Structure 15: 259–265. 17355861 10.1016/j.str.2007.02.001

[62] Barrick JE, Corbino KA, Winkler WC, Nahvi A, Mandal M, et al (2004) New RNA motifs suggest an expanded scope for riboswitches in bacterial genetic control. Proc Natl Acad Sci U S A 101: 6421–6426. 15096624 10.1073/pnas.0308014101 PMC404060

[63] Dann CE (2007) Structure and mechanism of a metal-sensing regulatory RNA. Cell 130: 878–892. 17803910 10.1016/j.cell.2007.06.051

[64] Fan P, Suri AK, Fiala R, Live D, Patel DJ (1996) Molecular recognition in the FMN-RNA aptamer complex. J Mol Biol 258: 480–500. 8642604 10.1006/jmbi.1996.0263

[65] Zimmermann GR, Jenison RD, Wick CL, Simorre JP, Pardi A (1997) Interlocking structural motifs mediate molecular discrimination by a theophylline-binding RNA. Nat Struct Biol 4: 644–649. 9253414 10.1038/nsb0897-644

[66] Flinders J, DeFina SC, Brackett DM, Baugh C, Wilson C, et al (2004) Recognition of planar and nonplanar ligands in the malachite green-RNA aptamer complex. Chembiochem 5: 62–72. 14695514 10.1002/cbic.200300701

[67] Guo F, Gooding AR, Cech TR (2004) Structure of the Tetrahymena ribozyme: base triple sandwich and metal ion at the active site. Mol Cell 16: 351–362. 15525509 10.1016/j.molcel.2004.10.003

[68] Xin Y, Laing C, Leontis NB, Schlick T (2008) Annotation of tertiary interactions in RNA structures reveals variations and correlations. RNA 14: 2465–2477. 18957492 10.1261/rna.1249208 PMC2590958

[69] Keating KS, Toor N, Pyle AM (2008) The GANC tetraloop: a novel motif in the group IIC intron structure. J Mol Biol 383: 475–481. 18773908 10.1016/j.jmb.2008.08.043 PMC2574657

[70] Butcher SE, Pyle AM (2011) The molecular interactions that stabilize RNA tertiary structure: RNA motifs, patterns, and networks. Acc Chem Res 44: 1302–1311. 21899297 10.1021/ar200098t

[71] Cate JH, Gooding AR, Podell E, Zhou K, Golden BL, et al (1996) RNA tertiary structure mediation by adenosine platforms. Science 273: 1696–1699. 8781229 10.1126/science.273.5282.1696

[72] Jang SB, Baeyens K, Jeong MS, SantaLucia J Jr, Turner D, et al (2004) Structures of two RNA octamers containing tandem G.A base pairs. Acta Crystallogr D Biol Crystallogr 60: 829–835. 15103128 10.1107/S0907444904003804

