# Supplementary figures and images for: Comparative Sequence and Structure Analysis Reveals the Conservation and Diversity of Nucleotide Positions and Their Associated Tertiary Interactions in the Riboswitches
Source: PLoS One. 2013 Sep 5;8(9):e73984. doi: 10.1371/journal.pone.0073984 (PMC3764141; doi:10.1371/journal.pone.0073984)

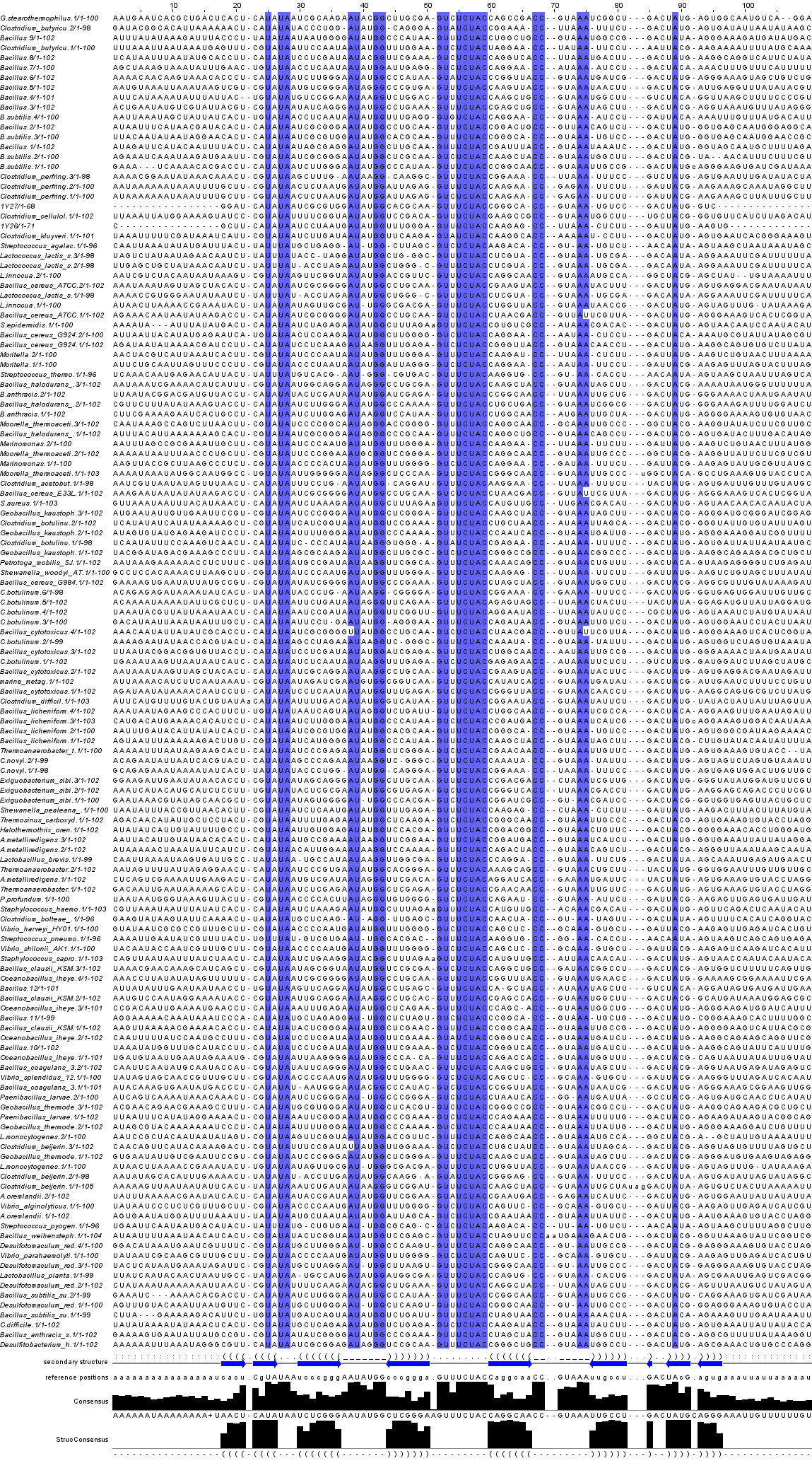

Supplement: Figure S1 — Rfam seed alignment for purine riboswitches. Blue shaded columns represent nucleotide positions that are more than 95% conserved. (PNG) [file pone.0073984.s001.png]

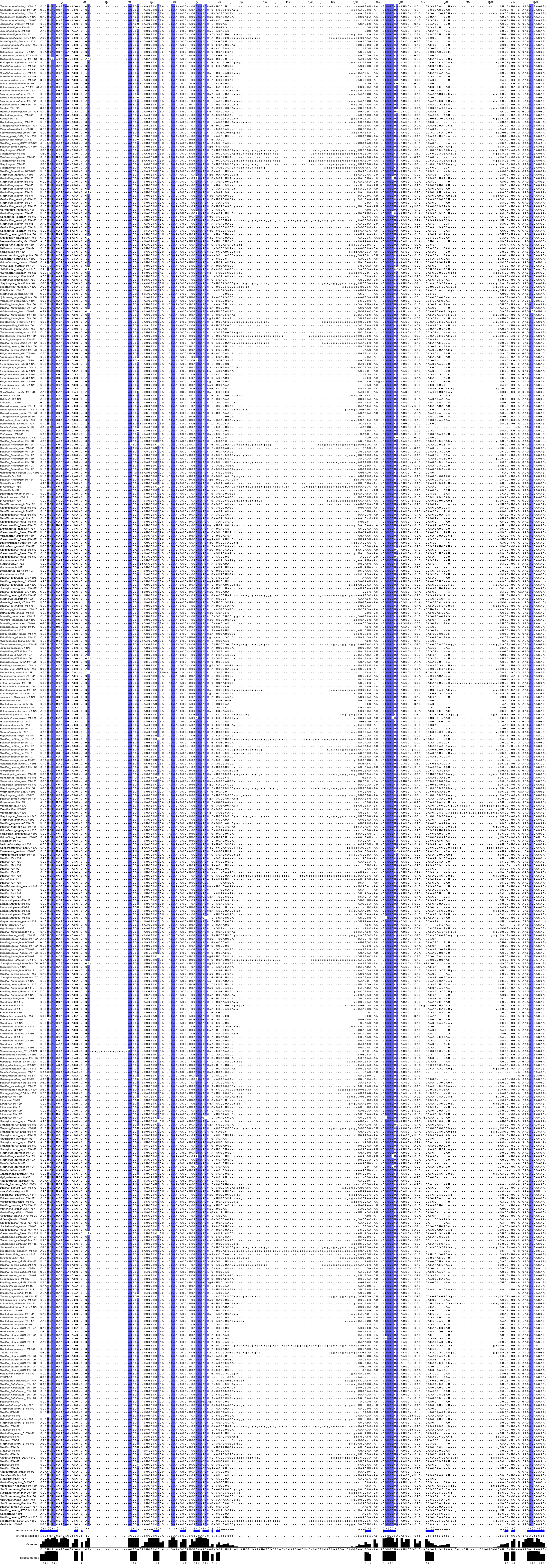

Supplement: Figure S2 — Rfam seed alignment for SAM-I riboswitches. Blue shaded columns represent nucleotide positions that are more than 95% conserved. (PNG) [file pone.0073984.s002.png]

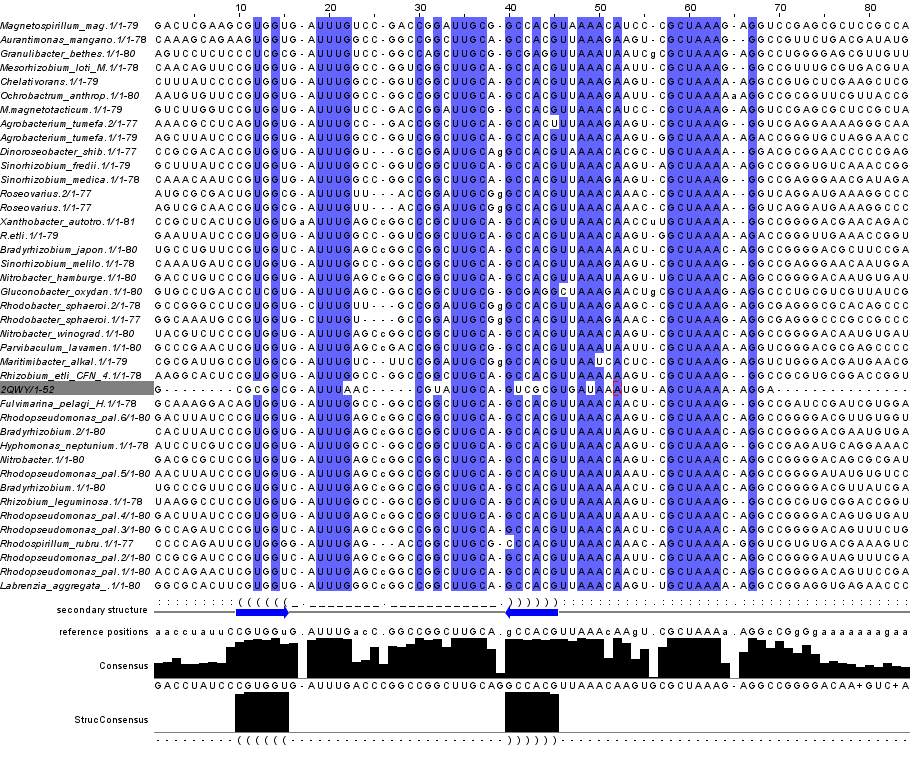

Supplement: Figure S3 — Rfam seed alignment for SAM-II riboswitches. Blue shaded columns represent nucleotide positions that are more than 95% conserved. (PNG) [file pone.0073984.s003.png]

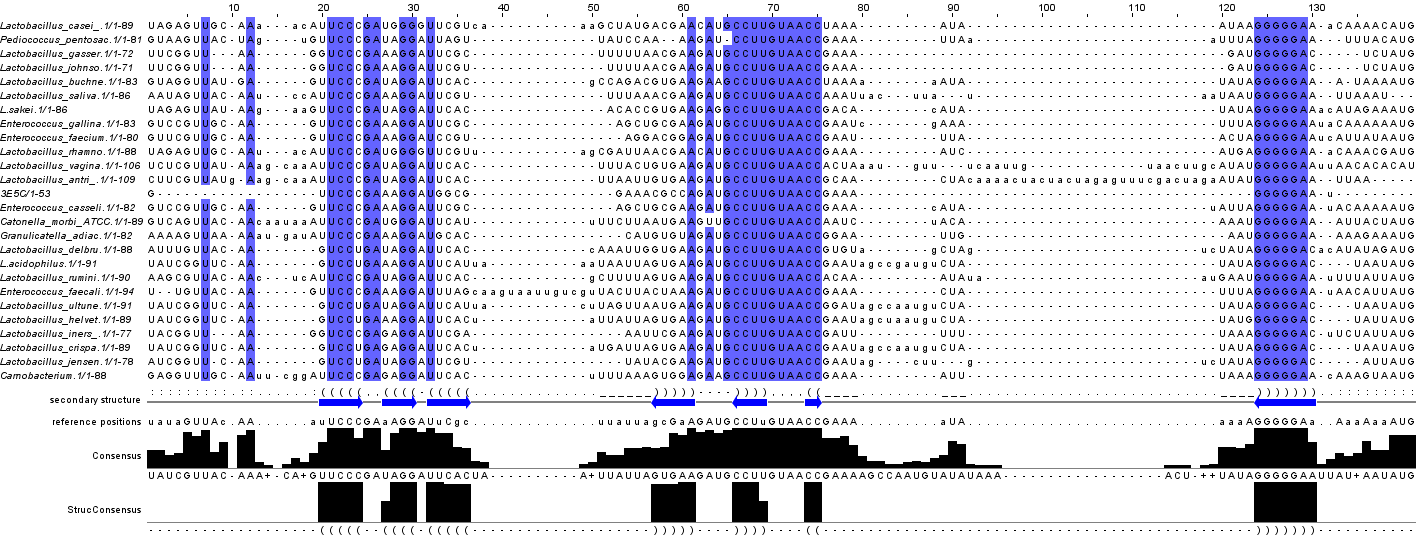

Supplement: Figure S4 — Rfam seed alignment for SAM-III riboswitches. Blue shaded columns represent nucleotide positions that are more than 95% conserved. (PNG) [file pone.0073984.s004.png]

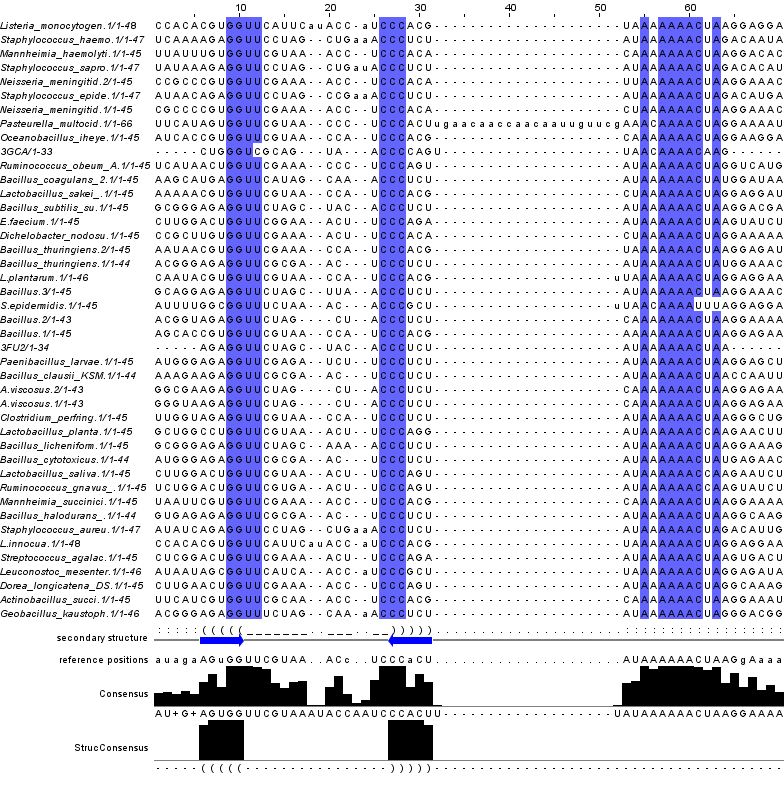

Supplement: Figure S5 — Rfam seed alignment for preQ1 riboswitches. Blue shaded columns represent nucleotide positions that are more than 95% conserved. (PNG) [file pone.0073984.s005.png]

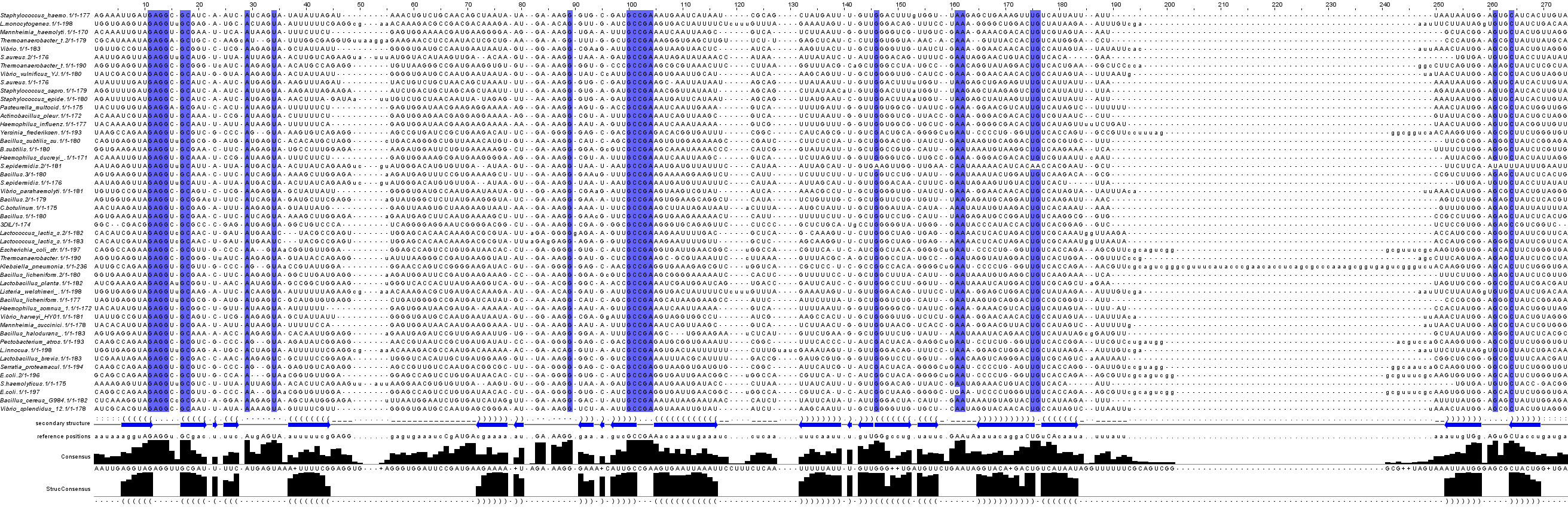

Supplement: Figure S6 — Rfam seed alignment for lysine riboswitches. Blue shaded columns represent nucleotide positions that are more than 95% conserved. (PNG) [file pone.0073984.s006.png]

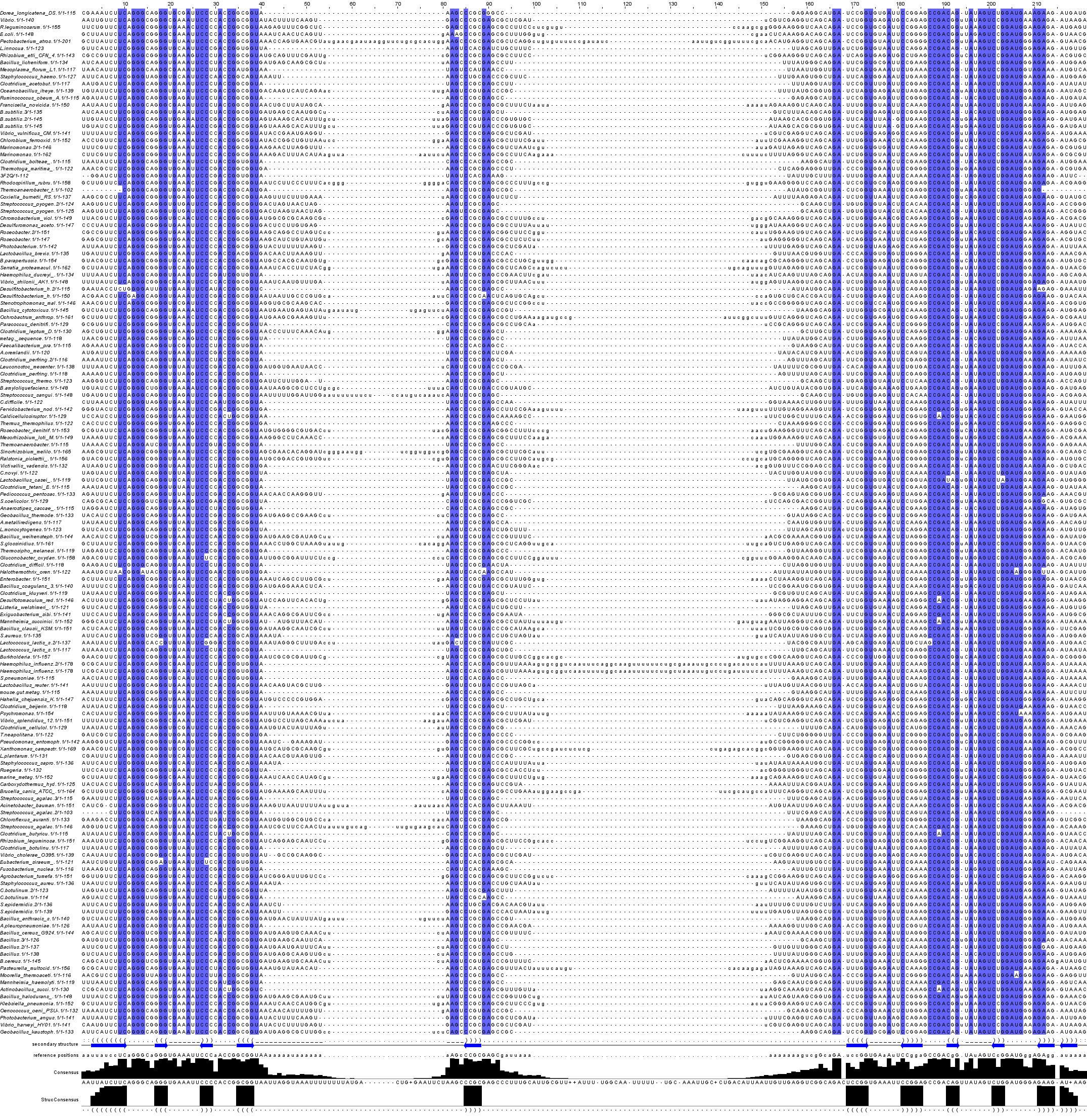

Supplement: Figure S7 — Rfam seed alignment for FMN riboswitches. Blue shaded columns represent nucleotide positions that are more than 95% conserved. (PNG) [file pone.0073984.s007.png]

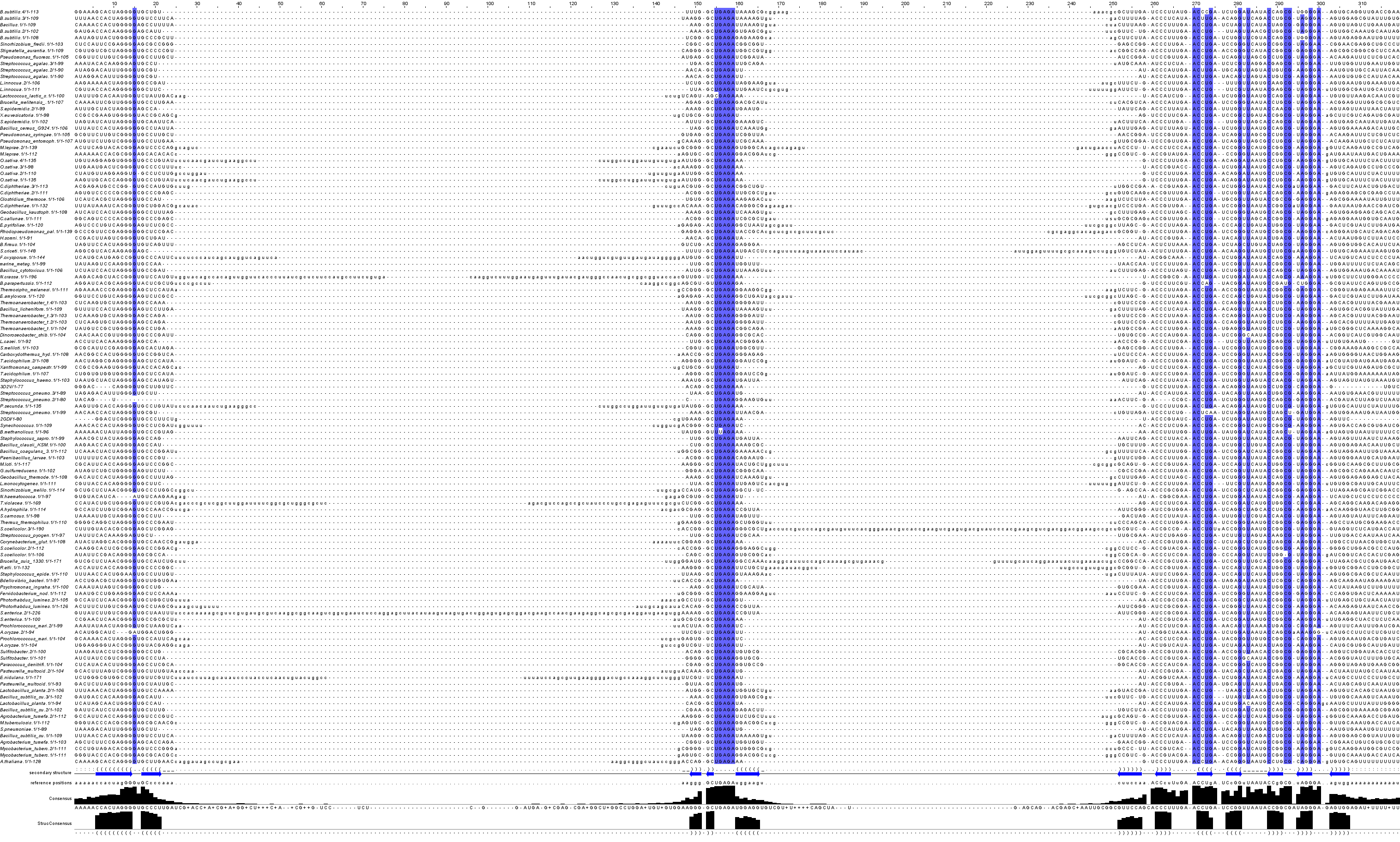

Supplement: Figure S8 — Rfam seed alignment for TPP riboswitches. Blue shaded columns represent nucleotide positions that are more than 95% conserved. (PNG) [file pone.0073984.s008.png]

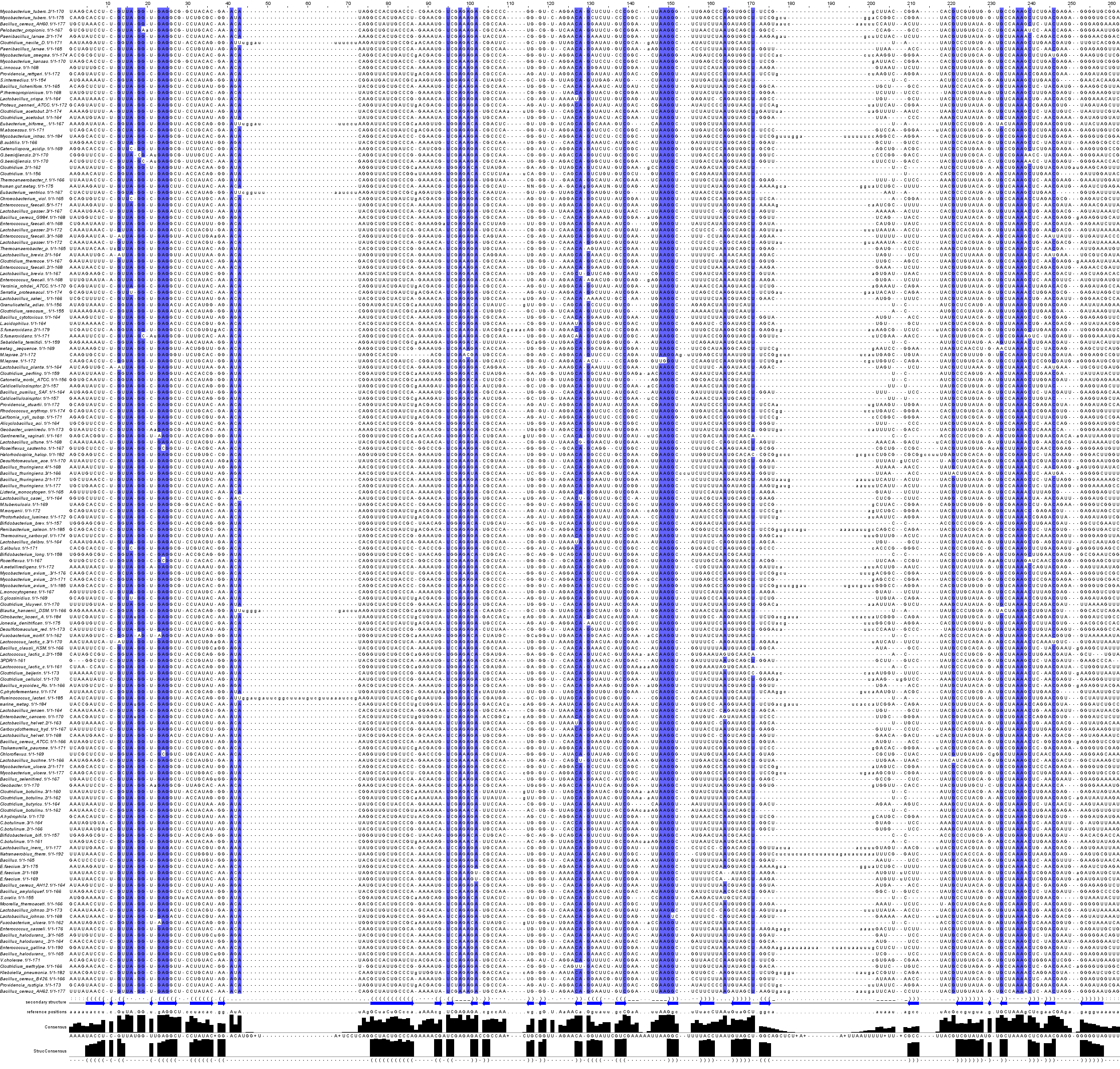

Supplement: Figure S9 — Rfam seed alignment for Mg2+ riboswitches. Blue shaded columns represent nucleotide positions that are more than 95% conserved. (PNG) [file pone.0073984.s009.png]

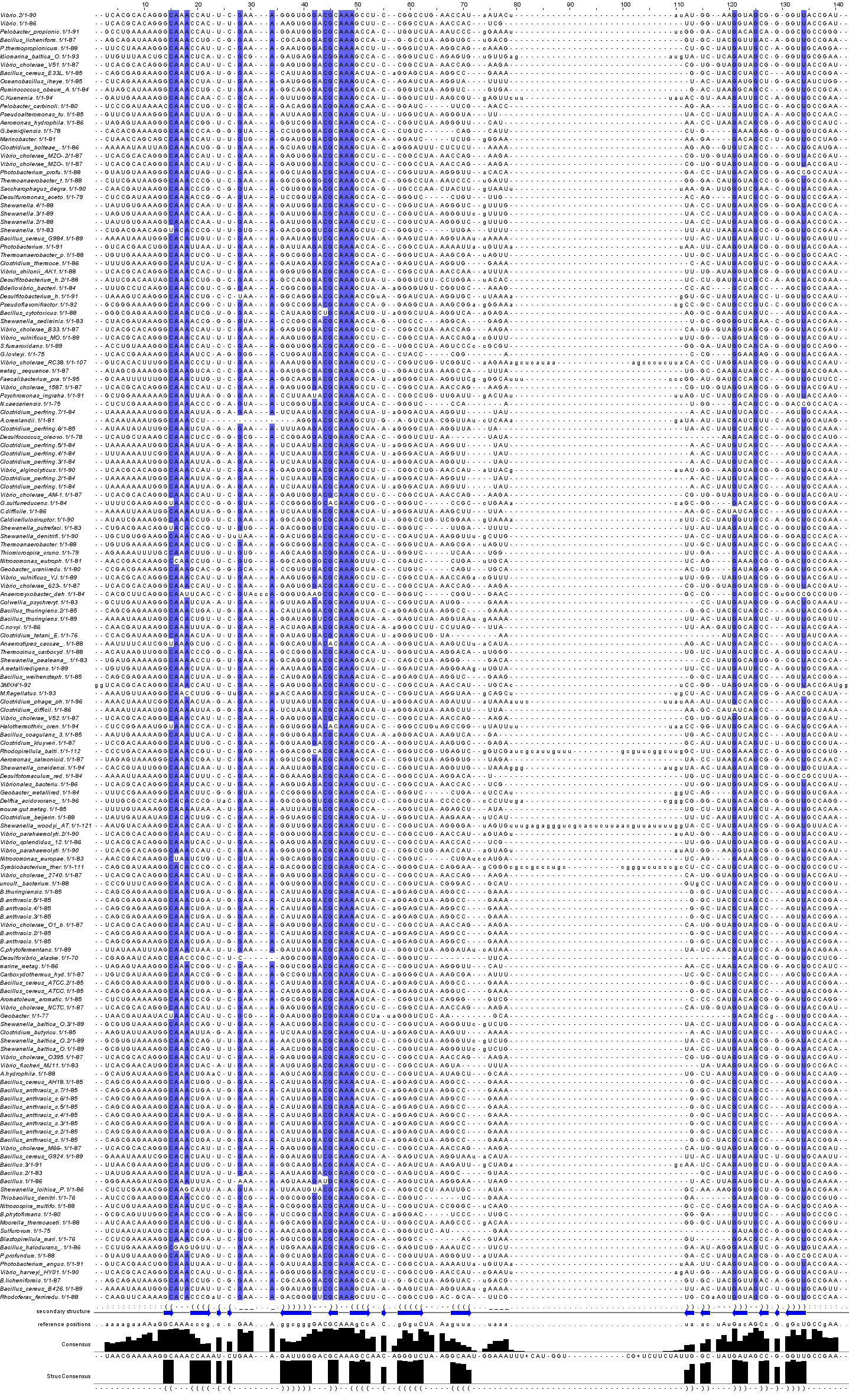

Supplement: Figure S10 — Rfam seed alignment for c-di-GMP riboswitches. Blue shaded columns represent nucleotide positions that are more than 95% conserved. (PNG) [file pone.0073984.s010.png]

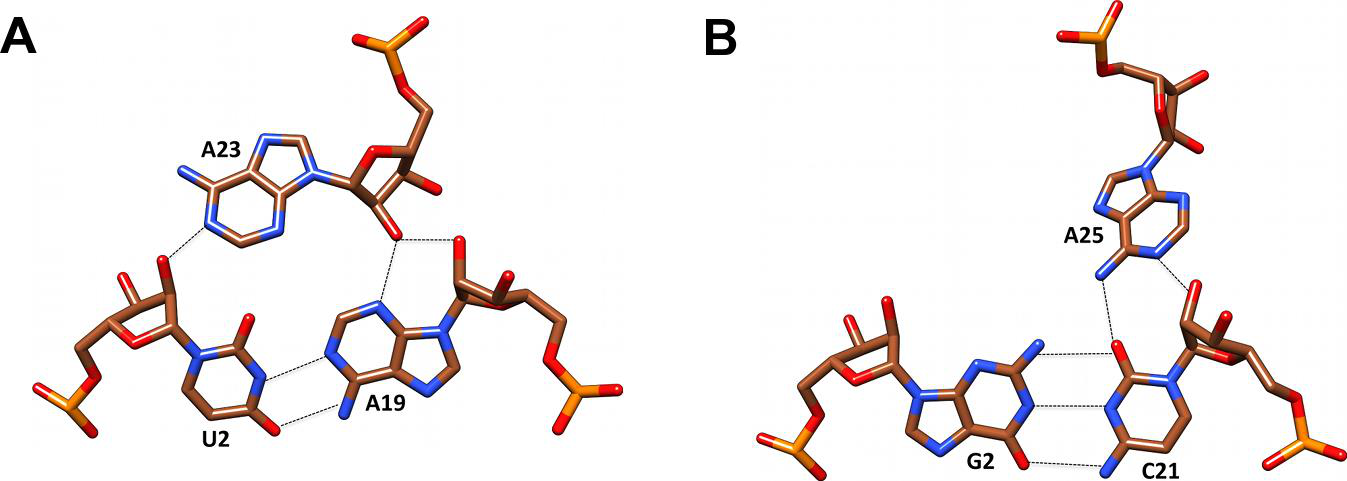

Supplement: Figure S11 — Pseudo-cis ribose zipper interaction previously annotated in the T. tencongensis preQ1 riboswitch. A) A23-A19-U2 A-minor in T. tencongensis preQ1 riboswitch [PDB: 3gca]. B) A25-C21-G2 base-triple in B. subtilis preQ1 riboswitch [PDB: 3fu2]. (TIFF) [file pone.0073984.s011.tiff]

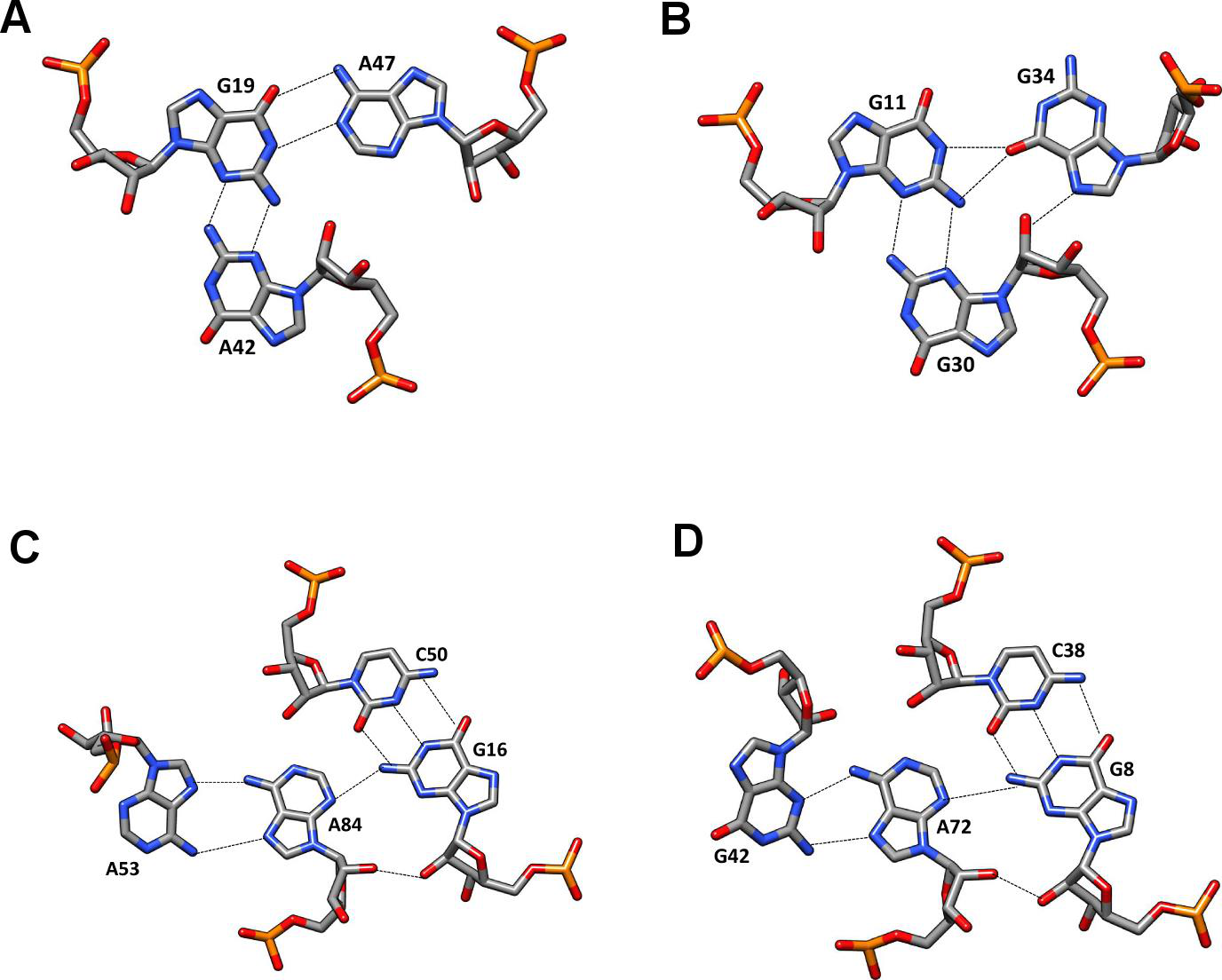

Supplement: Figure S12 — Differences in hydrogen bonding patterns between equivalent base interactions in TPP riboswitches. (A) G19-A42-A47 triple in E. coli TPP riboswitch [PDB: 2gdi] (B) G11-G30-G34 triple in A. thaliana riboswitch [PDB: 3d2v]. (C) A53-A84-G16-C50 quadruple in E. coli TPP riboswitch [PDB: 2gdi] (D) G42-A72-G8-C38 quadruple in A. thaliana TPP riboswitch [PDB: 3d2v]. (TIFF) [file pone.0073984.s012.tiff]
