# Supplementary material for: Comparative Sequence and Structure Analysis Reveals the Conservation and Diversity of Nucleotide Positions and Their Associated Tertiary Interactions in the Riboswitches
Source: PLoS One. 2013 Sep 5;8(9):e73984. doi: 10.1371/journal.pone.0073984 (PMC3764141; doi:10.1371/journal.pone.0073984)
Supplement: Table S1 — List of sequences and structures used for riboswitch sequence-structure comparisons. (DOC) [file pone.0073984.s013.doc]

**Table S1. Source of sequences and structures used for sequence-structure comparisons.**

|  | **Purine riboswitch** |  |
| --- | --- | --- |
| **Species** | **Taxonomy** | **Accession code/PDB ID** |
| *Bacillus licheniformis* *ATCC 14580* | Firmicutes; Bacilli | CP000002 |
| *Thermoanaerobacter tengcongensis* *MB4* | Firmicutes; Bacilli | AE0086991 |
| *Lactobacillus brevis* *ATCC 367* | Firmicutes; Bacilli | CP000416 |
| *Shewanella pealeana* ATCC 700345 | Proteobacteria; Gammaproteobacteria | CP000851 |
| *Vibrio parahaemolyticus* *RIMD 2210633* | Proteobacteria; Gammaproteobacteria | BA000032 |
| *Listeria monocytogenes* *strain EGD* | Firmicutes; Bacilli | AL591975 |
| *Staphlococcus aureus subsp. aureus str. Newman* | Firmicutes; Bacilli | AP009351 |
| *Halothermothrix orenii H 168* | Firmicutes; Clostridia | CP001098 |
| *Clostridium novyi NT* | Firmicutes; Clostridia | CP000382 |
| *Geobacillus denitrificans NG80-2* | Firmicutes; Bacilli | CP000557 |
| *Desulfotomaculum reducens MI-1* | Firmicutes; Clostridia | CP00612 |
| *Alkaliphilus oremlandii OhILAs* | Firmicutes; Clostridia | CP000853 |
| *Marinomonas sp. MED121 1099517007689* | Proteobacteria; Gammaproteobacteria | AANE01000012 |
| *Vibrio vulnificus* | Firmicutes; Bacilli | 1y26 |
| *Bacillus subtilis* | Firmicutes; Bacilli | 1y27 |
|  | **SAM-I riboswitch** |  |
| **Species** | **Taxonomy** | **Accession code/PDB ID** |
| *Petrotoga mobilis SJ95* | Thermotogae; Thermotogales | CP000879 |
| *Flavobacterium johsoniae UW101* | Bacteroidetes; Flavobacteria | CP000685 |
| *Geobacter lovleyi SZ* | Proteobacteria; Deltaproteobacteria | CP001089 |
| *Chloroflexus aurantiacus J-10-fl* | Chloroflexi; Chloroflexales | CP000909 |
| *Thermobifida fusca YX* | Actinobacteria; Actinobacteridae | CP000088 |
| *Acidobacterium capsulatum atcc 51196* | Acidobacteria; Acidobacteriales | CP001472 |
| *Chlorobium limicola DSM 245* | Chlorobi; Chlorobia | CP001097 |
| *Gloebacter violaceus PCC 7421* | Cyanobacteria; Gloeobacteria | BA000045 |
| *Fusobacterium nucleatum susbsp. nucleatum ATCC 25586* | Fusobacteria; Fusobacteriales | AE009951 |
| *Clostridium novyi NT* | Firmicutes; Clostridia | CP000382 |
| *Bacillus clausii KSM-K16* | Firmicutes, Bacilli | AP006627 |
| Methylacidiphilum infernorum V4 | Verrucomicrobia | CP000975 |
| Thermoanaerobacter sp. X514 | Firmicutes; Clostridia | CP000923 |
| *Staphylococcus aureus subsp. aureus COL* | Firmicutes, Bacilli | CP000046 |
| *Oceanobacillus iheyensis HTE831 DNA* | Firmicutes, Bacilli | BA000028 |
| *Thermoanaerobacter tengcongensis* | Firmicutes; Clostridia | 2gis |
|  | **SAM-II riboswitch** |  |
| **Species** | **Taxonomy** | **Accession code/PDB ID** |
| *Bradyyrhizobium japonicum USDA 110* | Proteobacteria; alphaproteobacteria | BA000040 |
| *Nitrobacter winogradskyi Nb-255* | Proteobacteria; alphaproteobacteria | CP000115 |
| *Parvibaculum lavamentivorans* | Proteobacteria; alphaproteobacteria | CP000774 |
| *Gluconobacter oxydans 621H* | Proteobacteria; alphaproteobacteria | CP000009 |
| *Fulvimarina pelagi HTCC2506 1100011000318* | Proteobacteria; alphaproteobacteria | AATP01000002 |
| *Mesorhizobium loti MAFF303099 DNA* | Proteobacteria; alphaproteobacteria | BA000012 |
| *Rhodopseudomonas palustris CGA009* | Proteobacteria; alphaproteobacteria | BX572607 |
| *Agrobacterium tumefaciens str. C58* | Proteobacteria; alphaproteobacteria | AE007869 |
| *Rhizobium etli* | Proteobacteria; alphaproteobacteria | AJ012295 |
| *Sinorhizobium fredii NGR234* | Proteobacteria; alphaproteobacteria | CP001389 |
| *Xanthobacter autotrophicus Py2* | Proteobacteria; alphaproteobacteria | CP000781 |
| *Rhodobacter sphaeroides ATCC 17029* | Proteobacteria; alphaproteobacteria | CP000577 |
| *Granulibacter bethesdensis CGDNIH1* | Proteobacteria; alphaproteobacteria | CP000394 |
| *Aurantimonas manganoxydans SI85-9A1 1099451004071* | Proteobacteria; alphaproteobacteria | AAPJ01000002 |
| Environmental sequence from Sargasso Sea metagenome | - | 2qwy |
|  | **SAM-III riboswitch** |  |
| **Species** | **Taxonomy** | **Accession code/PDB ID** |
| *Catonella morbi ATCC 51271* | Firmicutes; Clostridia | ACIL02000003 |
| *Carnobacterium sp. AT7 1101238000965* | Firmicutes; Bacilli; Lactobacillales | ABHH01000037 |
| *Granulicatella adiacens ATCC 49175* | Firmicutes; Bacilli; Lactobacillales | ACKZ01000012 |
| *Enterococcus faecalis ATCC 29200* | Firmicutes; Bacilli; Lactobacillales | ACHK01000062 |
| *Enterococcus faecium Com15* | Firmicutes; Bacilli; Lactobacillales | ACBD01000055 |
| *Enterococcus gallinarum EG2* | Firmicutes; Bacilli; Lactobacillales | ACAJ01000030 |
| *Enterococcus casseliflavus EC20* | Firmicutes; Bacilli; Lactobacillales | ACAO01000044 |
| *Lactobacillus jensenii 208-1* | Firmicutes; Bacilli; Lactobacillales | ADEX01000093 |
| *Lactobacillus crispatus JV-V01* | Firmicutes; Bacilli; Lactobacillales | ACKR01000001 |
| *Lactobacillus ultunensis DSM 16047* | Firmicutes; Bacilli; Lactobacillales | ACGU01000017 |
| *Lactobacillus delbrueckii subsp. bulgaricus ATCC 11842* | Firmicutes; Bacilli; Lactobacillales | CR954253 |
| *Lactobacillus vaginalis ATCC 49540* | Firmicutes; Bacilli; Lactobacillales | ACGV01000186 |
| *Lactobacillus acidophilus NCFM* | Firmicutes; Bacilli; Lactobacillales | CP000033 |
| *Pediococcus pentosaceus ATCC 25745* | Firmicutes; Bacilli; Lactobacillales | CP000422 |
| *Enterococcus faecalis* | Firmicutes; Bacilli; Lactobacillales | 3e5c |
|  | **PreQ1 riboswitch** |  |
| **Species** | **Taxonomy** | **Accession code/PDB ID** |
| *Arthrobacter viscous* | Actinobacteria; Actinobacteridae | AF022216 |
| *Streptococcus agalactiae NEM316* | Firmicutes; Bacilli | AL766846 |
| *Bacillus sp. B14905 1101159007471* | Firmicutes, Bacilli | AAXV01000007 |
| *Staphylococcus epidermidis RP62A* | Firmicutes, Bacilli | CP00029 |
| *Staphylococcus saprophyticus subsp. saprophyticus ATCC 15305 DNA* | Firmicutes, Bacilli | AP008934 |
| *Clostridium perfringens CPE str. F4969* | Firmicutes; Clostridia | ABDX01000003 |
| *Dorea longicatena DSM 13814* | Firmicutes; Clostridia | AAXB02000001 |
| *Ruminococcus obeum ATCC 29174* | Firmicutes; Clostridia | AAVO02000002 |
| *Bacillus clausii KSM-K16 DNA* | Firmicutes, Bacilli | AP006627 |
| *Lactobacillus plantarum WCFS1* | Firmicutes; Bacilli | AL935263 |
| *Neisseria meningitidis serogroup C FAM18* | Proteobacteria: Betaproteobacteria | AM421808 |
| *Actinobacillus succinogenes 130Z* | Proteobacteria: Gammaproteobacteria | CP000746 |
| *Mannheimia succiniciproducens MBEL55E* | Proteobacteria: Gammaproteobacteria | AEO16827 |
| *Bacillus subtilis* | Firmicutes; Bacilli | 3fu2 |
| *Thermoanaerobacter tengcongensis* | Firmicutes; Clostridia | 3gca |
|  | **Lysine riboswitch** |  |
| **Species** | **Taxonomy** | **Accession code/PDB ID** |
| *Bacillus licheniformis ATCC 14580* | Firmicutes; Bacilli | CP000002 |
| *Lactobacillus brevis ATCC 367* | Firmicutes; Lactobacillales | CO000416 |
| *Lactococcus lactis subsp. lactis II1403* | Firmicutes; Lactobacillales | AE005176 |
| *Thermoanaebacter sp. X514* | Firmicutes; Clostridia | CP000923 |
| *Serratia proteamaculans 568* | Proteobacteria; Gammaproteobacteria | CP000826 |
| *Vibrio vulnificus YJ016* | Proteobacteria; Gammaproteobacteria | BA000037 |
| *Haemophilus influenzae F3031* | Proteobacteria; Gammaproteobacteria | FQ670178 |
| Clostridium botulinum C str. Eklund | Firmicutes; Clostridia | ABDQ01000005 |
| *Staphylococcus aureus strain 8325-4* | Firmicutes; Bacilli | AF306669 |
| *Listeria monocytogenes strain EGD* | Firmicutes; Bacilli | AL591976 |
| *Klebsiella pneumoniae subsp. pneumoniae MGH 78578* | Proteobacteria; Gammaproteobacteria | CP000647 |
| *Actinobacillus pleuropneumoniae L20 serotype 5b* | Proteobacteria; Gammaproteobacteria | CP000569 |
| *Mannheimia succiniciproducens MBEL55E* | Proteobacteria; Gammaproteobacteria | AE016827 |
| *Escherichia. coli* | Proteobacteria; Gammaproteobacteria | X00008 |
| *Thermotoga maritima* | Thermotogae; Thermotogaceae | 3dil |
|  | **FMN riboswitch** |  |
| **Species** | **Taxonomy** | **Accession code/PDB ID** |
| *Xanthomonas campestris pv. campestris str. ATCC 33913* | Proteobacteria; Gammaproteobacteria | AE00892 |
| *Enterobacter sp. 638* | Proteobacteria; Gammaproteobacteria | CP000653 |
| *Streptomyces coelicolor A3* | Actinobacteria; Actinobacteridae | AL939108 |
| *Chloroflexus aurantiacus J-10-fl* | Chloroflexi; Chloroflexales | CP000909 |
| *Thermus thermophilus HB27* | Deinococcus-Thermus | AE017221 |
| *Bacillus licheniformis DSM 13* | Firmicutes; Bacilli | AE017333 |
| *Rhizobium etli CFN 42* | Proteobacteria; Alphaproteobacteria | CP000133 |
| *Rhodospirillum rubrum ATCC 11170* | Proteobacteria; Alphaproteobacteria | CP000230 |
| *Burkholderia sp. 383* | Proteobacteria; Betaproteobacteria | CP000152 |
| *Thermotoga maritima MSB8* | Thermotogae; Thermotogaceae | AE000512 |
| *Fusobacterium nucleatum subsp. nucleatum ATCC 25586* | Fusobacteria; Fusobacteriales | AE009951 |
| *Clostridium botulinum F str. Langeland* | Firmicutes; Clostridia | CP000728 |
| *Lactobacillus casei ATCC 334* | Firmicutes; Lactobacillales | CP000423 |
| *Chlorobium ferrooxidans DSM 13031 ctg60* | Chlorobi; Chlorobia | AASE01000003 |
| *Fusobacterium nucleatum* | Fusobacteria; Fusobacteriales | 3f2q |
|  | **TPP riboswitch** |  |
| ***Species*** | **Taxonomy** | **Accession code/PDB ID** |
| *Thermoplasma acidophilum* | Archaea; Euryarchaeota | AL445064 |
| *Corynebacterium diphteriae gravis NCTC13129* | Actinobacteria; Actinobacteridae | BX248356 |
| *Mycobacterium tuberculosis H37Rv* | Actinobacteria; Actinobacteridae | BX842573 |
| *Prochlorococcus marinus MIT9313* | Cyanobacteria; Prochlorales | BX548175 |
| *Thermus thermophilus HB27* | Deinococcus-Thermus; Deinococci | AE017221 |
| *Bacillus licheniformis ATCC 14580* | Firmicutes; Bacilli | CP000002 |
| *Clostridium thermocellum ATCC 27405* | Firmicutes; Clostridia | CP000568 |
| *Agrobacterium tumefaciens str. C58* | Proteobacteria; alphaproteobacteria | AE007870 |
| *Bdellovibrio bacteriovorus* | Proteobacteria; deltaproteobacteria | BX842649 |
| *Xanthomonas campestris pv. campestris str. 8004* | Proteobacteria; gammaproteobacteria | CP00050 |
| *Aspergillus oryzae* | Eukaryota; fungi | AF217503 |
| *Oryza sativa Japonica* | Eukaryota; viridiplantae | AK119882 |
| *Lactobacillus casei* | Firmicutes; Lactobacillales | AF159589 |
| *Escherichia. coli* | Proteobacteria; gammaproteobacteria | 2hol |
| *Arabidopsis thaliana* | Eukaryota; viridiplantae | 3d2v |
|  | **Mg^2+^ riboswitch** |  |
| **Species** | **Taxonomy** | **Accession code/PDB ID** |
| *Mycobacterium tuberculosis F11,* | Actinobacteria; Actionobacteridae | CP000717 |
| *Roseiflexus castenholzii DSM 13941* | Chloroflexi; Chloroflexales | CP000804 |
| *Bacillus cereus G9842* | Firmicutes; Bacilli | CP001186 |
| *Clostridium botulinum A3. str Loch Maree, complete genome* | Firmicutes; Clostridia | CP000962 |
| *Carboxydothermus hydrogenoformans Z-2901* | Firmicutes; Clostridia | CP000141 |
| *Enterococcus faecalis V583* | Firmicutes; Lactobacillales | AE016830 |
| *Chromobacterium violaceum ATCC 12472* | Proteobacteria; Betaproteobacteria | AE016825 |
| *Geobacter uraniireducens Rf4* | Proteobacteria; Deltaproteobacteria | CP000698 |
| *Syntrophobacter fumaroxidans MPOB* | Proteobacteria; Deltaproteobacteria | CP000478 |
| *Aeromonas hydrophila flg operon* | Proteobacteria; Gammaproteobacteria | AY129558 |
| *Serratia proteamaculans 568* | Proteobacteria; Gammaproteobacteria | CP000826 |
| *Lactococcus lactis subsp. lactisII1403* | Firmicutes; Lactobacillales | AE005176 |
| *Listeria monocytogenes HCC23* | Firmicutes; Bacilli | CP001175 |
| *Lactobacillus casei ATCC 334* | Firmicutes; Lactobacillales | CP000423 |
| *Bacillus subtilis* | Firmicutes; Bacilli | 3pdr |
|  | **c-di-GMP riboswitch** |  |
| ***Species*** | **Taxonomy** | **Accession code/PDB ID** |
| *Bacillus anthracis. str. CDC684* | Firmicutes; Bacilli | CP001215 |
| *Clostridium novyi NT* | Firmicutes; Clostridia | CP000382 |
| *Kuenenia stuttgartiensis genome fragment KUSD_T* | Planctomycetes; Planctomycetia | CT573072 |
| *Burkholderia phytofirmans PsJN chromosome 1* | Proteobacteria; Betaproteobacteria | CP001052 |
| *Aeromonas hydrophilla subsp. hydrophilla ATCC 7966* | Proteobacteria; Gammaproteobacteria | CP000462 |
| *Sulfurovum sp. NBC37-1* | Proteobacteria; Epsilonproteobacteria | AP009179 |
| *Nitrosomonas eutropha C91* | Proteobacteria; Betaproteobacteria | CP000450 |
| *Thiobacillus denitrificans ATCC 25259* | Proteobacteria; Betaproteobacteria | CP000116 |
| *Thermoanaerobacter pseudethanolicus ATCC 33223* | Firmicutes; Clostridia | CP000924 |
| *Shewanella sp. W3-18-1* | Proteobacteria; Gammaproteobacteria | CP000503 |
| *Geobacter lovleyi SZ* | Proteobacteria; Deltaproteobacteria | CP001089 |
| *Psychromonas ingrahamii 37* | Proteobacteria; Gammaproteobacteria | CP000510 |
| *Syntrophobacter fumaroxidans MPOB* | Proteobacteria; Deltaproteobacteria | CP000478 |
| *Halothermothrix orenii H 168* | Firmicutes; Clostridia | CP001098 |
| *Vibrio cholerae* | Proteobacteria; Gammaproteobacteria | 3mxh |
