# Supplementary material for: Comparative Sequence and Structure Analysis Reveals the Conservation and Diversity of Nucleotide Positions and Their Associated Tertiary Interactions in the Riboswitches
Source: PLoS One. 2013 Sep 5;8(9):e73984. doi: 10.1371/journal.pone.0073984 (PMC3764141; doi:10.1371/journal.pone.0073984)
Supplement: Table S2 — List of annotated tertiary motifs in riboswitches. (DOC) [file pone.0073984.s014.doc]

**Table S2.** List of annotated tertiary interactions in riboswitches

**1) Purine riboswitch** (Reference structure PDB ID = **1y26**)

- A-minor

| **ID** |  | **Residue 1** |  |  | **Residue 2** |  |  | **Residue 3** |  |
| --- | --- | --- | --- | --- | --- | --- | --- | --- | --- |
|  | **Seq** | **Seq_Pos** | **Chain** | **Seq** | **Seq_Pos** | **Chain** | **Seq** | **Seq_Pos** | **Chain** |
| 1 | A | 33 | X | G | 37 | X | C | 61 | X |

- Base triple

| **ID** |  | **Residue 1** |  |  | **Residue 2** |  |  | **Residue 3** |  |
| --- | --- | --- | --- | --- | --- | --- | --- | --- | --- |
|  | **Seq** | **Seq_Pos** | **Chain** | **Seq** | **Seq_Pos** | **Chain** | **Seq** | **Seq_Pos** | **Chain** |
| 1 | A | 23 | X | G | 46 | X | C | 53 | X |
| 2 | A | 66 | X | G | 38 | X | C | 60 | X |

- Loop-loop interaction

| **ID** | **Strand 1** | | **Strand 2** | |
| --- | --- | --- | --- | --- |
|  | **Seq_Pos** | **Chain** | **Seq_Pos** | **Chain** |
| 1 | 33-38 | X | 60-65 | X |

**2) SAM-I riboswitch** (Reference structure PDB ID = **2gis**)

- A-minor

| **ID** |  | **Residue 1** |  |  | **Residue 2** |  |  | **Residue 3** |  |
| --- | --- | --- | --- | --- | --- | --- | --- | --- | --- |
|  | **Seq** | **Seq_Pos** | **Chain** | **Seq** | **Seq_Pos** | **Chain** | **Seq** | **Seq_Pos** | **Chain** |
| 1 | G | 11 | A | C | 44 | A | G | 58 | A |
| 2 | A | 12 | A | C | 59 | A | G | 43 | A |
| 3 | A | 36 | A | C | 31 | A | G | 21 | A |

- Base triple

| **ID** |  | **Residue 1** |  |  | **Residue 2** |  |  | **Residue 3** |  |
| --- | --- | --- | --- | --- | --- | --- | --- | --- | --- |
|  | **Seq** | **Seq_Pos** | **Chain** | **Seq** | **Seq_Pos** | **Chain** | **Seq** | **Seq_Pos** | **Chain** |
| 1 | A | 62 | A | G | 23 | A | C | 29 | A |

- Kink-turn

| **ID** |  | **Strand I** |  |  | **Strand 2** |  |
| --- | --- | --- | --- | --- | --- | --- |
|  | **Seq** | | **Chain** | **Seq** | | **Chain** |
| 1 | 30-38 | | A | 17-22 | | A |

- Pseudoknot

| **ID** | **Seq_Pos** | **Chain** |
| --- | --- | --- |
| 1 | 25-28, 65-68 | A |

- Ribose zipper

| **ID** |  | **Strand I** |  |  | **Strand 2** |  |
| --- | --- | --- | --- | --- | --- | --- |
|  | **Seq** | **Seq_Pos** | **Chain** | **Seq** | **Seq_Pos** | **Chain** |
| 1 | GA | 11,12 | A | GC | 43,44 | A |

**3)** **SAM-II riboswitch** (Reference structure PDB ID = **2qwy**)

- A-minor

| **ID** |  | **Residue 1** |  |  | **Residue 2** |  |  | **Residue 3** |  |
| --- | --- | --- | --- | --- | --- | --- | --- | --- | --- |
|  | **Seq** | **Seq_Pos** | **Chain** | **Seq** | **Seq_Pos** | **Chain** | **Seq** | **Seq_Pos** | **Chain** |
| 1 | A | 37 | A | G | 5 | A | C | 27 | A |
| 2 | A | 36 | A | C | 4 | A | G | 28 | A |

- Base triple

| **ID** |  | **Residue 1** |  |  | **Residue 2** |  |  | **Residue 3** |  |
| --- | --- | --- | --- | --- | --- | --- | --- | --- | --- |
|  | **Seq** | **Seq_Pos** | **Chain** | **Seq** | **Seq_Pos** | **Chain** | **Seq** | **Seq_Pos** | **Chain** |
| 1 | G | 8 | A | G | 42 | A | C | 23 | A |
| 2 | U | 11 | A | A | 45 | A | U | 21 | A |
| 3 | U | 12 | A | A | 46 | A | U | 20 | A |

- Ribose-zipper

| **ID** |  | **Strand I** |  |  | **Strand 2** |  |
| --- | --- | --- | --- | --- | --- | --- |
|  | **Seq** | **Seq_Pos** | **Chain** | **Seq** | **Seq_Pos** | **Chain** |
| 1 | CG | 27,28 | A | AA | 36,37 | A |

- Pseudoknot – The whole structure adopts a H-type pseudoknot

**4) SAM-III riboswitch** (Reference structure PDB ID = **3e5c**)

- Base-triple

| **ID** |  | **Residue 1** |  |  | **Residue 2** |  |  | **Residue 3** |  |
| --- | --- | --- | --- | --- | --- | --- | --- | --- | --- |
|  | **Seq** | **Seq_Pos** | **Chain** | **Seq** | **Seq_Pos** | **Chain** | **Seq** | **Seq_Pos** | **Chain** |
| 1 | A | 27 | A | G | 71 | A | G | 66 | A |
| 2 | A | 73 | A | G | 90 | A | C | 25 | A |

**5) PreQ1 riboswitch** (Reference structure PDB ID = **3fu2**)

- Base triple

| **ID** |  | **Residue 1** |  |  | **Residue 2** |  |  | **Residue 3** |  |
| --- | --- | --- | --- | --- | --- | --- | --- | --- | --- |
|  | **Seq** | **Seq_Pos** | **Chain** | **Seq** | **Seq_Pos** | **Chain** | **Seq** | **Seq_Pos** | **Chain** |
| 1 | A | 16 | A | G | 11 | A | C | 31 | A |
| 2 | A | 28 | A | G | 5 | A | C | 18 | A |

- Pseudoknot – The whole structure adopts a H-type psudoknot

**6) Lysine riboswitch** (Reference structure PDB ID = **3dil**)

- A-minor

| **ID** |  | **Residue 1** |  |  | **Residue 2** |  |  | **Residue 3** |  |
| --- | --- | --- | --- | --- | --- | --- | --- | --- | --- |
|  | **Seq** | **Seq_Pos** | **Chain** | **Seq** | **Seq_Pos** | **Chain** | **Seq** | **Seq_Pos** | **Chain** |
| 1 | A | 81 | A | G | 14 | A | C | 78 | A |
| 2 | A | 51 | A | C | 41 | A | G | 54 | A |

- Base-triple

| **ID** |  | **Residue 1** |  |  | **Residue 2** |  |  | **Residue 3** |  |
| --- | --- | --- | --- | --- | --- | --- | --- | --- | --- |
|  | **Seq** | **Seq_Pos** | **Chain** | **Seq** | **Seq_Pos** | **Chain** | **Seq** | **Seq_Pos** | **Chain** |
| 1 | A | 23 | A | G | 69 | A | A | 127 | A |
| 2 | G | 141 | A | A | 162 | A | G | 163 | A |

- Loop-loop interaction

| **ID** | **Strand 1** | | **Strand 2** | |
| --- | --- | --- | --- | --- |
|  | **Seq_Pos** | **Chain** | **Seq_Pos** | **Chain** |
| 1 | 44-49 | A | 95-100 | A |

- Loop-receptor interaction

| **ID** | **Loop** | | **Receptor** | |
| --- | --- | --- | --- | --- |
|  | **Seq_Pos** | **Chain** | **Seq_Pos** | **Chain** |
| 1 | 125-129 | A | 68-69, 23-24 | A |

**7) FMN riboswitch** (Reference structure PDB ID = **3f2q**)

- A-minor

| **ID** |  | **Residue 1** |  | **Residue 2** | | | **Residue 3** | | |
| --- | --- | --- | --- | --- | --- | --- | --- | --- | --- |
|  | **Seq** | **Seq_Pos** | **Chain** | **Seq** | **Seq_Pos** | **Chain** | **Seq** | **Seq_Pos** | **Chain** |
| 1 | A | 63 | X | G | 41 | X | C | 82 | X |
| 2 | A | 104 | X | G | 33 | X | C | 46 | X |
| 3 | G | 11 | X | G | 84 | X | C | 31 | X |
| 4 | G | 62 | X | G | 32 | X | C | 83 | X |

- Base-triple

| **ID** |  | **Residue 1** |  | **Residue 2** | | | **Residue 3** | | |
| --- | --- | --- | --- | --- | --- | --- | --- | --- | --- |
|  | **Seq** | **Seq_Pos** | **Chain** | **Seq** | **Seq_Pos** | **Chain** | **Seq** | **Seq_Pos** | **Chain** |
| 1 | G | 12 | X | G | 93 | X | C | 30 | X |

- Loop-loop interaction

| **ID** | **Strand 1** | | **Strand 2** | |
| --- | --- | --- | --- | --- |
|  | **Seq_Pos** | **Chain** | **Seq_Pos** | **Chain** |
| 1 | 18-22 | X | 90 | X |
| 2 | 69-73 | X | 38 | X |

- Ribose-zipper

| **ID** |  | **Strand I** |  |  | **Strand 2** |  |
| --- | --- | --- | --- | --- | --- | --- |
|  | **Seq** | **Seq_Pos** | **Chain** | **Seq** | **Seq_Pos** | **Chain** |
| 1 | CG | 46,47 | X | GA | 103,104 | X |

**8) Prokaryotic TPP riboswitch** (Reference structure PDB ID: **2gdi**)

- A-minor

| **ID** |  | **Residue 1** |  | **Residue 2** | | | **Residue 3** | | |
| --- | --- | --- | --- | --- | --- | --- | --- | --- | --- |
|  | **Seq** | **Seq_Pos** | **Chain** | **Seq** | **Seq_Pos** | **Chain** | **Seq** | **Seq_Pos** | **Chain** |
| 1 | A | 56 | X | G | 17 | X | C | 49 | X |
| 2 | A | 84 | X | C | 50 | X | G | 16 | X |
| 3 | A | 70 | X | G | 37 | X | C | 22 | X |
| 4 | A | 41 | X | G | 18 | X | C | 48 | X |

- Base triple

| **ID** |  | **Residue 1** |  | **Residue 2** | | | **Residue 3** | | |
| --- | --- | --- | --- | --- | --- | --- | --- | --- | --- |
|  | **Seq** | **Seq_Pos** | **Chain** | **Seq** | **Seq_Pos** | **Chain** | **Seq** | **Seq_Pos** | **Chain** |
| 1 | G | 19 | X | G | 42 | X | A | 47 | X |

- Loop-receptor interaction

| **ID** | **Loop** | | **Receptor** | |
| --- | --- | --- | --- | --- |
|  | **Seq_Pos** | **Chain** | **Seq_Pos** | **Chain** |
| 1 | 68-71 | X | 21-22, 37-38 | X |

- Ribose zipper

| **ID** |  | **Strand I** |  |  | **Strand 2** |  |
| --- | --- | --- | --- | --- | --- | --- |
|  | **Seq** | **Seq_Pos** | **Chain** | **Seq** | **Seq_Pos** | **Chain** |
| 1 | AU | 70,71 | X | GC | 21,22 | X |

**9) Eukaryotic TPP riboswitch** (Reference structure PDB ID: **3d2v**)

- A-minor

| **ID** |  | **Residue 1** |  | **Residue 2** | | | **Residue 3** | | |
| --- | --- | --- | --- | --- | --- | --- | --- | --- | --- |
|  | **Seq** | **Seq_Pos** | **Chain** | **Seq** | **Seq_Pos** | **Chain** | **Seq** | **Seq_Pos** | **Chain** |
| 1 | A | 44 | A | G | 9 | A | C | 37 | A |
| 2 | A | 72 | A | C | 38 | A | G | 8 | A |
| 3 | A | 58 | A | G | 25 | A | C | 14 | A |
| 4 | A | 29 | A | G | 10 | A | C | 36 | A |

- Base triple

| **ID** |  | **Residue 1** |  | **Residue 2** | | | **Residue 3** | | |
| --- | --- | --- | --- | --- | --- | --- | --- | --- | --- |
|  | **Seq** | **Seq_Pos** | **Chain** | **Seq** | **Seq_Pos** | **Chain** | **Seq** | **Seq_Pos** | **Chain** |
| 1 | G | 11 | A | G | 30 | A | G | 34 | A |
| 1 | A | 43 | A | U | 47 | A | A | 68 | A |

- Loop-receptor interaction

| **ID** | **Loop** | | **Receptor** | |
| --- | --- | --- | --- | --- |
|  | **Seq_Pos** | **Chain** | **Seq_Pos** | **Chain** |
| 1 | 56-59 | A | 13-14, 25-26 | A |

- Ribose zipper

| **ID** |  | **Strand I** |  |  | **Strand 2** |  |
| --- | --- | --- | --- | --- | --- | --- |
|  | **Seq** | **Seq_Pos** | **Chain** | **Seq** | **Seq_Pos** | **Chain** |
| 1 | AU | 58,59 | A | GC | 13,14 | A |

**10) Magnesium riboswitch** (Reference structure PDB ID: **3pdr**)

- A-minor

| **ID** |  | **Residue 1** |  | **Residue 2** | | | **Residue 3** | | |
| --- | --- | --- | --- | --- | --- | --- | --- | --- | --- |
|  | **Seq** | **Seq_Pos** | **Chain** | **Seq** | **Seq_Pos** | **Chain** | **Seq** | **Seq_Pos** | **Chain** |
| 1 | A | 88 | X | G | 151 | X | C | 33 | X |
| 2 | A | 117 | X | G | 84 | X | C | 57 | X |
| 3 | A | 71 | X | G | 22 | X | C | 163 | X |
| 4 | A | 155 | X | G | 107 | X | C | 99 | X |
| 5 | A | 105 | X | G | 73 | X | C | 68 | X |

- Base triple

| **ID** |  | **Residue 1** |  |  | **Residue 2** |  |  | **Residue 3** |  |
| --- | --- | --- | --- | --- | --- | --- | --- | --- | --- |
|  | **Seq** | **Seq_Pos** | **Chain** | **Seq** | **Seq_Pos** | **Chain** | **Seq** | **Seq_Pos** | **Chain** |
| 1 | U | 24 | X | G | 100 | X | A | 106 | X |
| 2 | A | 46 | A | U | 138 | A | A | 139 | A |
| 3 | C | 35 | X | U | U34 | X | U | 150 | X |

- Loop-receptor interaction

| **ID** | **Loop** | | **Receptor** | |
| --- | --- | --- | --- | --- |
|  | **Seq_Pos** | **Chain** | **Seq_Pos** | **Chain** |
| 1 | 69-72 | X | 22, 163-164 | X |

- Ribose zipper

| **ID** |  | **Strand I** |  |  | **Strand 2** |  |
| --- | --- | --- | --- | --- | --- | --- |
|  | **Seq** | **Seq_Pos** | **Chain** | **Seq** | **Seq_Pos** | **Chain** |
| 1 | AA | 70,71 | X | CG | 163,164 | X |

**11) c-di-GMP riboswitch** (Reference structure PDB ID: **3mxh**)

- A-minor

| **ID** |  | **Residue 1** |  | **Residue 2** | | | **Residue 3** | | |
| --- | --- | --- | --- | --- | --- | --- | --- | --- | --- |
|  | **Seq** | **Seq_Pos** | **Chain** | **Seq** | **Seq_Pos** | **Chain** | **Seq** | **Seq_Pos** | **Chain** |
| 1 | A | 35 | R | G | 79 | R | C | 59 | R |
| 2 | A | 49 | R | G | 45 | R | C | 22 | R |
| 3 | A | 34 | R | A | 78 | R | U | 60 | R |

- Base triple

| **ID** |  | **Residue 1** |  |  | **Residue 2** |  |  | **Residue 3** |  |
| --- | --- | --- | --- | --- | --- | --- | --- | --- | --- |
|  | **Seq** | **Seq_Pos** | **Chain** | **Seq** | **Seq_Pos** | **Chain** | **Seq** | **Seq_Pos** | **Chain** |
| 1 | A | 95 | R | G | 14 | R | C | 93 | R |

- Loop-receptor interaction

| **ID** | **Loop** | | **Receptor** | |
| --- | --- | --- | --- | --- |
|  | **Seq_Pos** | **Chain** | **Seq_Pos** | **Chain** |
| 1 | 32-35 | R | 59-60, 78-79 | R |

- Ribose zipper

| **ID** |  | **Strand I** |  |  | **Strand 2** |  |
| --- | --- | --- | --- | --- | --- | --- |
|  | **Seq** | **Seq_Pos** | **Chain** | **Seq** | **Seq_Pos** | **Chain** |
| 1 | AA | 33,34 | R | CU | 59,60 | R |
