# Supplementary material for: Comparative Sequence and Structure Analysis Reveals the Conservation and Diversity of Nucleotide Positions and Their Associated Tertiary Interactions in the Riboswitches
Source: PLoS One. 2013 Sep 5;8(9):e73984. doi: 10.1371/journal.pone.0073984 (PMC3764141; doi:10.1371/journal.pone.0073984)
Supplement: Table S3 — List of highly conserved nucleotides in riboswitches. (DOC) [file pone.0073984.s015.doc]

**Table S3.** List of nucleotides that have more than 95% sequence conservation in the Rfam seed alignments for the 10 riboswitch classes. These nucleotides are grouped according to the seven annotated tertiary motifs or others if they are not found in any of the tertiary interactions.

| **Riboswitch Class** | **Reference Structure PDB ID** | **Highly Conserved Nucleotides** |
| --- | --- | --- |
| Purine | 1y26 | **A-minor:** A33, G37, C61  **Base-triple**: A23, G46, C53, G38, C60, A66  **Loop-loop interaction**: A33, U34, G37, G38, C60, C61, A65  **Others**: U20, U22, U47, U49. C50, U51, A52, A76 |
| SAM-I | 2gis | **A-minor**: G11, A12, G21, C31, A36, G43, C44, C58  **Base-triple**: G23, C29  **Kink-turn**: G19, A20, G21, C31, G35, A36  **Ribose-zipper**: G11, A12, G43, C44  **Others**: U4, A6, U7, A45, A46, C47, G56, U57, A61, A87, U88 |
| SAM-II | 2qwy | **A-minor**: C4, G28, A37  **Base-triple**: U11, U12, U20, U21, C23, G42, A45, A46  **Ribose-zipper**: G28, A37  **Others**: G6, U10, A13, C16, U18, G22, U26, G30, U31, A33, U34, A35, C43, U44, A47, G50 |
| SAM-III | 3e5c | **Base-triple**: A27, G71, A73, G90  **Others**: U22, C23, C24, G26, G30, G31, C67, C68, U69, U70, U72, A74, C75, C76, G88, G89, G91, G92, A93 |
| preQ1 | 3fu2 | **Base-triple**: G5, C18, A28, C31  **Others**: G4, U6, U7, C17, C19, A25, A27, A29, A30, A33 |
| Lysine | 3dil | **A-minor**: G14, C78, A81  **Base-triple**: A23, G69, A127, G141, G163  **Loop-receptor interaction**: A23, G69, A126, A127  **Others**: G9, A10, G11, G12, C15, U28, G77, C79, G80, G114, U140, C166 |
| FMN | 3f2q | **A-minor**: G11, C31, G32, G62, A63, C82, C83, C84, A104  **Base-triple**: G12, G93  **Loop-loop interaction:** G19, A22, U69, A73, A90  **Ribose-zipper**: G47, A104  **Others**: G10,G16, G17, U24, C25, C26, A29, G36, U37, A40, C43, A48, G68, C76, A85, C86, G88, A92, U94, C95, G97, G98, A99, U100, G101, G105, A106 |
| TPP | 2gdi | **A-minor**: A41, A56, A84  **Base-triple**: G19, G42  **Loop-receptor interaction**: U68  **Others**: U39, G40, A43, C57, U59, G60, G66, U68, C73, C77, G78, A80, G82 |
| Mg^2+^ | 3pdr | **A-minor**: G22, C99, G107, G151, A155  **Base-triple**: U24, A46, A106  **Loop-receptor interaction**: G22, A70, A72  **Ribose-zipper**: A70  **Others**: A25, G26, G27, G29, A30, G31, G32, A44, U67, A74, A90, G91, A105, G108, U125, G141, A156, C158 |
| cyclic di-GMP | 3mxh | **A-minor**: C22, A35, G45, A49, C59, G79  **Loop-receptor interaction**:G32, A35, C59, G79  **Others**: A23, A25, G42, C44, A47, A48, G83, U90 |
